# A secreted citrus protease cleaves an outer membrane protein of the Huanglongbing pathogen

**DOI:** 10.1073/pnas.2528641123

**Published:** 2026-04-07

**Authors:** Alexander J. McClelland, Bin Hu, Yuantao Xu, Xiaodong Fang, Chunxia Wang, Benjamin L. Koch, Amelia H. Lovelace, Eva Hawara, Yuanchun Wang, Zhiqian Pang, Agustina De Francesco, Suzanne P. van Wier, Andrew M. Beekman, Amit Levy, Nian Wang, Renier A. L. van der Hoorn, Qiang Xu, Wenbo Ma

**Affiliations:** ^a^The Sainsbury Laboratory, University of East Anglia, Norwich NR4 7UH, United Kingdom; ^b^National Key Laboratory for Germplasm Innovation and Utilization of Horticultural Crops, Huazhong Agricultural University, Wuhan 430070, China; ^c^Citrus Research and Education Center, Department of Plant Pathology, Microbiology and Cell Science, University of Florida/Institute of Food and Agricultural Sciences, Lake Alfred, FL 33850; ^d^Department of Microbiology and Plant Pathology, University of California Riverside, Riverside, CA 92521; ^e^School of Chemistry, Pharmacy and Pharmacology, University of East Anglia, Norwich NR4 7TJ, United Kingdom; ^f^Plant Chemetics Laboratory, Department of Biology, University of Oxford, Oxford OX1 3RB, United Kingdom

**Keywords:** citrus greening disease, plant innate immunity, bacterial pathogens, Candidatus Liberibacter, plant-pathogen interactions

## Abstract

Plants secrete a variety of proteases as a defense response during infection by microbial pathogens. However, the relationship between their catalytic activities and antimicrobial functions remains largely unknown. Particularly, few biologically relevant substrates of these proteases have been identified. Huanglongbing (HLB) has been a major threat to the citrus industry worldwide. The HLB-associated bacterium, “*Candidatus* Liberibacter asiaticus” (Las), was previously shown to deploy an inhibitor of papain-like cysteine proteases (PLCPs) to promote disease in citrus. In this study, we identified an outer membrane protein (OMP) of Las, LasOMP1, as a substrate of the citrus PLCP *Cs*RD21a. LasOMP1 is one of the most highly expressed genes in Las. *Cs*RD21a cleaves LasOMP1 and produces cleaved peptide products, which could be detected in vitro and in HLB-diseased citrus plants. We found that *Cs*RD21a targets the N-terminal portion of LasOMP1, potentially at an extracellular loop region. Importantly, transgenic sweet orange overexpressing *Cs*RD21a showed reduced Las populations and improved plant growth, highlighting that engineering this protease is a promising strategy to enhance HLB resistance in citrus. Together, our work reveals a pathogen-derived substrate of plant PLCPs and suggests bacterial OMPs may be direct targets of plant defense.

## Significance

Huanglongbing (HLB) is the most devastating disease of citrus with no resistance having been identified in commercial cultivars. Previous work implicated papain-like cysteine proteases (PLCPs) as an important hub of defense in citrus; however, their precise role in HLB resistance remained unclear. Here, we identify and characterize an outer membrane protein (OMP) from the HLB-associated bacterium as a substrate of the citrus PLCP RD21a. We demonstrate a specific cleavage of the bacterial OMP by citrus RD21a, which may impair pathogen growth and/or activate plant immunity. Importantly, overexpression of RD21a enhances HLB resistance in sweet oranges. This work identifies OMPs as substrates of plant PLCPs and provides insights into protease defense functions.

Plant hosts deploy a myriad of hydrolytic enzymes to suppress pathogen virulence. Among them, many proteases are secreted and have established functions in plant defense (1–3). For example, the papain-like cysteine protease (PLCP) RD21a from *Arabidopsis thaliana* and its homologs in rice and wheat contribute to defense against a range of pathogens, including nematodes, protists, viruses, and fungi (4). In line with this defense role, evolutionarily distant plant pathogens commonly deploy virulence proteins, known as effectors, that possess protease inhibitor activities as a counterdefense strategy (1, 5). For example, the oomycete pathogen *Phytophthora infestans* produces several PLCP-targeting effectors to promote disease (6–9). In turn, proteases can be co-opted by plant immune receptors to function as guarded decoys that trigger receptor-mediated defense upon inhibition by pathogen effectors (10). However, specific substrates of the proteases that directly contribute to their defense functions remain poorly understood.

Extracellular proteases can cleave both endogenous substrates and pathogen-derived substrates. Cleavage of endogenous substrates may release immunogenic damage-associated molecular patterns (DAMPs) or activate other proteases with defense functions (11, 12). On the other hand, cleavage of pathogen-derived substrates may directly lead to antimicrobial activities and/or release pathogen-associated molecular patterns (PAMPs) that activate cell surface receptor-mediated defense, called PAMP-triggered immunity (PTI). To date, only a handful of pathogen-derived substrates of secreted plant proteases have been identified. PC2, a small, secreted protein from the oomycete pathogen *Phytophthora infestans*, is cleaved by a host subtilase, releasing a peptide that triggers cell death and thus restricts pathogen growth (13). Soybean secretes an aspartic protease that degrades a *Phytophthora sojae* cell wall-degrading enzyme, XEG1, thus contributing to resistance (14). Arabidopsis aspartic proteases cleave a periplasmic protein, MucD, from the bacterial pathogen *Pseudomonas syringae*, thus directly inhibiting bacterial growth (15). Last, the plant subtilase SBT5.2 balances immune responses by both releasing and degrading PAMPs to activate and suppress defense, respectively (16, 17). The identification of protease substrates offers a unique opportunity to uncover mechanisms of plant immunity and provides important insights into engineering strategies for disease resistance in crops.

Huanglongbing (HLB), also known as citrus greening, is the most devastating citrus disease to which all commercial citrus varieties are susceptible (18, 19). HLB is primarily caused by gram-negative bacteria belonging to “*Candidatus* Liberibacter” species, including “*Ca.* Liberibacter asiaticus” (Las), “*Ca.* Liberibacter americanus” (Lam), and “*Ca.* Liberibacter africanus” (Laf), with Las being the most widespread and impactful to the citrus industry. “*Ca.* Liberibacter” species are specialized, obligate bacteria transmitted by insect vectors, called psyllids, which deposit the bacteria into the plant phloem during feeding. In addition to the HLB-associating bacteria, “*Ca.* L. solanacearum” (Lso) causes disease in tomato, potato, pepper, and carrot (18, 20, 21). These bacteria have undergone significant genome loss compared to their pathogenic and nonpathogenic relatives in the Rhizobiaceae family, resulting in their inability to be cultured in vitro (22, 23). Each of these pathogens colonizes the plant phloem tissue, where mechanisms of plant defense responses are poorly understood.

Our previous characterization of Las effectors identified Sec-delivered effector 1 (SDE1) as a PLCP-inhibitor, implicating a role of PLCPs in phloem-based defense responses (24). SDE1 interacts with proteases from multiple PLCP subfamilies via their conserved protease domains, resulting in a decrease in their proteolytic activity in vitro and in planta (24). Transgenic citrus expressing SDE1 exhibited enhanced susceptibility to Las and accelerated HLB disease progression, indicating a correlation between PLCP inhibition and defense suppression (25). Furthermore, citrus PLCPs are induced during Las infection, consistent with their role as important nodes of defense in the phloem (24, 26, 27). Yet the mechanisms underlying PLCP defense functions are unclear.

In this work, we investigated the roles of PLCPs in citrus defense during Las infection. We found that *Citrus sinensis* RD21a (*Cs*RD21a) specifically interacts with an outer membrane protein (OMP) of Las named OMP1. This interaction leads to the cleavage of LasOMP1 near its N-terminal region. LasOMP1 is among the most highly expressed genes in Las and is also conserved across Liberibacter species, suggesting a critical role in bacterial survival and/or host colonization. Furthermore, LasOMP1 cleavage by PLCPs resulted in induced expression of a defense-related gene, leading to the possibility that an immunogenic peptide may be produced through this process that activates plant immunity. Finally, we generated transgenic citrus plants that overexpress *Cs*RD21a, which showed enhanced resistance to HLB as reflected by decreased Las populations and improved plant growth. Together, these results shed light on the mechanisms of protease-based defense in an economically important disease and reveal bacterial OMPs as targets of plant immune system.

## Results

### Citrus PLCPs Interact with a Highly Expressed Outer Membrane Protein of Las.

**Fig. 1.**
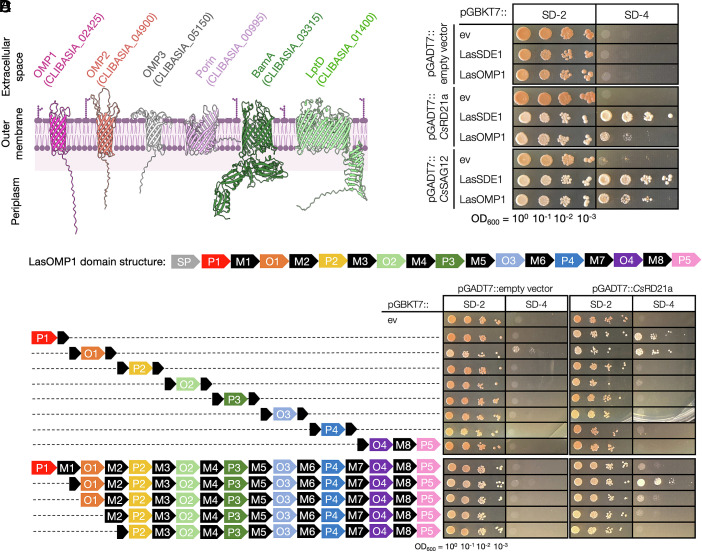
The citrus PLCP *Cs*RD21a interacts with the N-terminus of LasOMP1. (*A*) AlphaFold3 structural models of all Las OMPs without their signal peptides. All models were predicted with pTM scores between 0.65 and 0.84. The membrane image was sourced from BioRender.com. (*B*) Yeast-two-hybrid assay examining interactions between citrus PLCPs *Cs*RD21a and *Cs*SAG12 and Las membrane proteins. LasSDE1 and empty vectors (ev) served as positive and negative controls, respectively. SD-2 medium lacks leucine and tryptophan and was used to select the cotransformed colonies. One colony was serially diluted from OD_600_ = 1.0 and plated for each cotransformation in yeast on both SD-2 and SD-4, which lacks leucine, tryptophan, adenine, and histidine. Growth on SD-4 indicates protein–protein interaction. All proteins were expressed without their signal peptides. (*C*) LasOMP1 domain architecture (not to scale) predicted by OMPdb and DeepTMHMM. SP = signal peptide, P = periplasmic, M = membrane-bound, and O = outside/extracellular. (*D*) Yeast-two-hybrid assay examining interactions of *Cs*RD21a with LasOMP1 truncations. Dashed lines indicate deleted portions of LasOMP1, and the signal peptide of LasOMP1 was excluded.

The exposed surface of the outer membrane (OM) of gram-negative bacteria is enriched in *β*-barrel outer membrane proteins (OMPs) and lipopolysaccharide (28). OMPs provide structural support to the bacterial cell and form channels for the passage of molecules in and out of the cell. OMPs contain an N-terminal signal peptide (SP) that facilitates their secretion into the periplasm followed by an OM-embedded transmembrane *β*-barrel domain which contains periplasmic and extracellular loops (29). As the dominant proteins on the bacterial cell surface (28), OMPs are exposed directly to the plant phloem during infection. We therefore hypothesized they may be targets of plant defense mechanisms. Using a hidden Markov model (HMM)-based approach (30, 31), we predicted six Las *β*-barrel OMPs from a total of 1,017 proteins encoded in the Las genome (strain psy62) and modeled their protein structures without the N-terminal SPs using AlphaFold 3 (Fig. 1*A*). We further used DeepTMHMM topology predictions (31) to determine the number of the transmembrane *β*-strands in each predicted OMP. Two 16-stranded *β*-barrel OMPs were annotated as “porin” or “BamA,” and one 26-stranded β-barrel OMP was annotated as “LptD,” based on orthology to characterized OMPs in other bacteria. In addition, three eight-stranded *β*-barrel OMPs were identified and subsequently named OMP1, OMP2, and OMP3, respectively.

To investigate potential interactions between citrus PLCPs and Las OMPs, we performed yeast two-hybrid assays using *Cs*RD21a and *Cs*SAG12 as the baits. We found that both *Cs*RD21a and *Cs*SAG12 interacted with OMP1, hereafter LasOMP1, but not with the other OMPs or an inner-membrane protein YajC (Fig. 1*B* and *SI* *Appendix*, Fig. S1*A*). The relatively weaker interactions observed between the citrus PLCPs and LasOMP1 compared to LasSDE1 may indicate a less stable association, possibly due to proteolytic activity of the PLCPs against LasOMP1. Using publicly available transcriptomic data (32, 33), we found that LasOMP1 is the most highly expressed OMP and one of the most highly expressed Las genes overall during the colonization of both citrus and psyllids (*SI* *Appendix*, Fig. S1*B*). This expression profile suggests that LasOMP1 may have important biological functions in bacterial growth and host colonization.

Using OMPdb and DeepTMHMM (31, 34), we generated a LasOMP1 domain map (Fig. 1*C*). Following the N-terminal SP, LasOMP1 is predicted to contain five periplasmic (*P*) domains, eight membrane-bound (*M*) domains, and four extracellular/outside (*O*) domains. This topology, in which the OMP1 C-terminus is exposed to the periplasmic side of the outer membrane, is consistent with the structural requirement of the BamA-mediated OMP insertion into the OM (35–38). Using this domain architecture as a guide, we generated eight truncations, dividing LasOMP1 at each of the first seven membrane-bound domains (M1 to M7). The truncations were then tested for interaction with *Cs*RD21a via yeast-two-hybrid. Two truncations near the N-terminus of LasOMP1, P1 and O1, were still able to interact with *Cs*RD21a, although the O1 truncate exhibited a weak autoactivation (Fig. 1*D*). To further confirm *Cs*RD21a association with the N-terminal region of LasOMP1, a series of truncated mutants were generated and tested for interactions with *Cs*RD21a. The results showed that the interaction with *Cs*RD21a was abolished only when both P1 and O1 domains were removed (Fig. 1*D*), demonstrating that the N-terminal P1 and O1 regions of LasOMP1 are both sufficient and necessary for interaction with host PLCP *Cs*RD21a.

### *Cs*RD21a Cleaves LasOMP1 in a Semi-in vitro Assay.

**Fig. 2.**
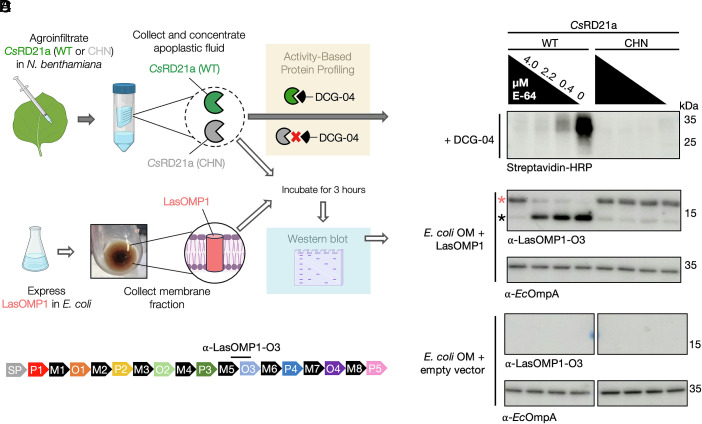
*Cs*RD21a cleaves LasOMP1 in a semi-in vitro assay. (*A*) Workflow of the semi-in vitro and activity-based protein profiling (ABPP) assays made using BioRender.com. (*B*) Western blot detection of wild-type (WT) or catalytically dead (CHN) *Cs*RD21a in apoplastic fluid (AF) using ABPP, where a concentration gradient of the cysteine protease inhibitor E-64 was incubated with the AF prior to DCG-04 labeling. Western blot detection of DCG-04-bound proteases was performed using Streptavidin-HRP. (*C*) Cleavage of LasOMP1 by *Cs*RD21a. E-64- or DMSO-treated AF containing *Cs*RD21a WT or the CHN mutant was incubated with *E. coli* membrane fractions containing LasOMP1. *Ec*OmpA was used as a loading control. Both OMPs were detected by Western blotting using protein-specific antibodies. The band representing full-length LasOMP1 is marked with a pink asterisk, and cleaved product is marked with a black asterisk. Membrane fractions extracted from *E. coli* carrying the empty vector were used as the negative control. (*D*) Domain structure of LasOMP1 showing the binding site of the α-LasOMP1-O3 antibody.

To assess whether LasOMP1 is a substrate of *Cs*RD21a, we established a semi-in vitro protease cleavage assay (Fig. 2*A*). It has been previously demonstrated that Arabidopsis RD21a undergoes both N- and C-terminal processing during its activation and secretion (39, 40). Therefore, we opted to express *Cs*RD21a without a tag and confirmed its enzymatic activity using activity-based protein profiling (ABPP). As a negative control, a catalytic mutant of *Cs*RD21a, *Cs*RD21a^CHN^, was generated in which each of its three catalytic residues (C163, H299, and N319) were replaced with alanine. Full-length *Cs*RD21a and *Cs*RD21a^CHN^ were expressed via *Agrobacterium*-mediated transient expression in *Nicotiana benthamiana,* and their secreted proteins (lacking an SP) were collected from apoplastic fluid (AF) and incubated with the biotinylated ABPP cysteine protease probe DCG-04 (41). Using streptavidin-HRP, we detected clear DCG-04 labeling signals of *Cs*RD21a between 25 and 35 kDa in leaves expressing the wild-type protein (Fig. 2*B*), corresponding to active proteoforms that have undergone removal of the autoinhibitory prodomain (*SI* *Appendix*, Fig. S2). This signal decreased in the presence of the cysteine protease inhibitor E-64 in a dosage-dependent manner, confirming that the enzymatic activity detected was indeed from *Cs*RD21a (Fig. 2*B*). As expected, no ABPP signal was observed in samples expressing *Cs*RD21a^CHN^.

To test if LasOMP1 can be cleaved by *Cs*RD21a, the AF extracts containing the active or catalytic mutant of *Cs*RD21a were incubated with LasOMP1 produced in *E. coli* (Fig. 2*A*). To maximize LasOMP1 secretion, we replaced its native SP with the *E. coli* OmpA SP. Membrane fractions of *E. coli* were collected from LasOMP1-expressing cells using cells carrying the empty vector as a control. *Ec*OmpA was detected with an *Ec*OmpA-specific antibody, confirming the enrichment of bacterial OMPs in the samples (Fig. 2*C*). LasOMP1 was detected using a LasOMP1-specific antibody (α-LasOMP1-O3), which was raised against the third extracellular loop (O3) (Fig. 2*D*). These bacterial membrane protein extracts were incubated with *Cs*RD21a-containing AF for 3 h at room temperature. LasOMP1 appeared in its mature, secreted form (lacking the SP) (~19.2 kDa) when incubated with wild-type *Cs*RD21a and a high concentration of E-64 (4.0 *µ*M) (Fig. 2*C*). However, a lower band below 15 kDa appeared, with a significant reduction of full-length LasOMP1 protein, in the presence of low concentrations of E-64. E-64 did not affect LasOMP1 stability when incubated with *Cs*RD21a^CHN^, suggesting that LasOMP1 is proteolytically cleaved only by wild-type *Cs*RD21a. Unlike LasOMP1, *Ec*OmpA was not affected by *Cs*RD21a (Fig. 2*C*), further demonstrating that the cleavage of LasOMP1 by *Cs*RD21a was specific.

### *Cs*RD21a Interacts with OMP1 Homologs in Liberibacter.

**Fig. 3.**
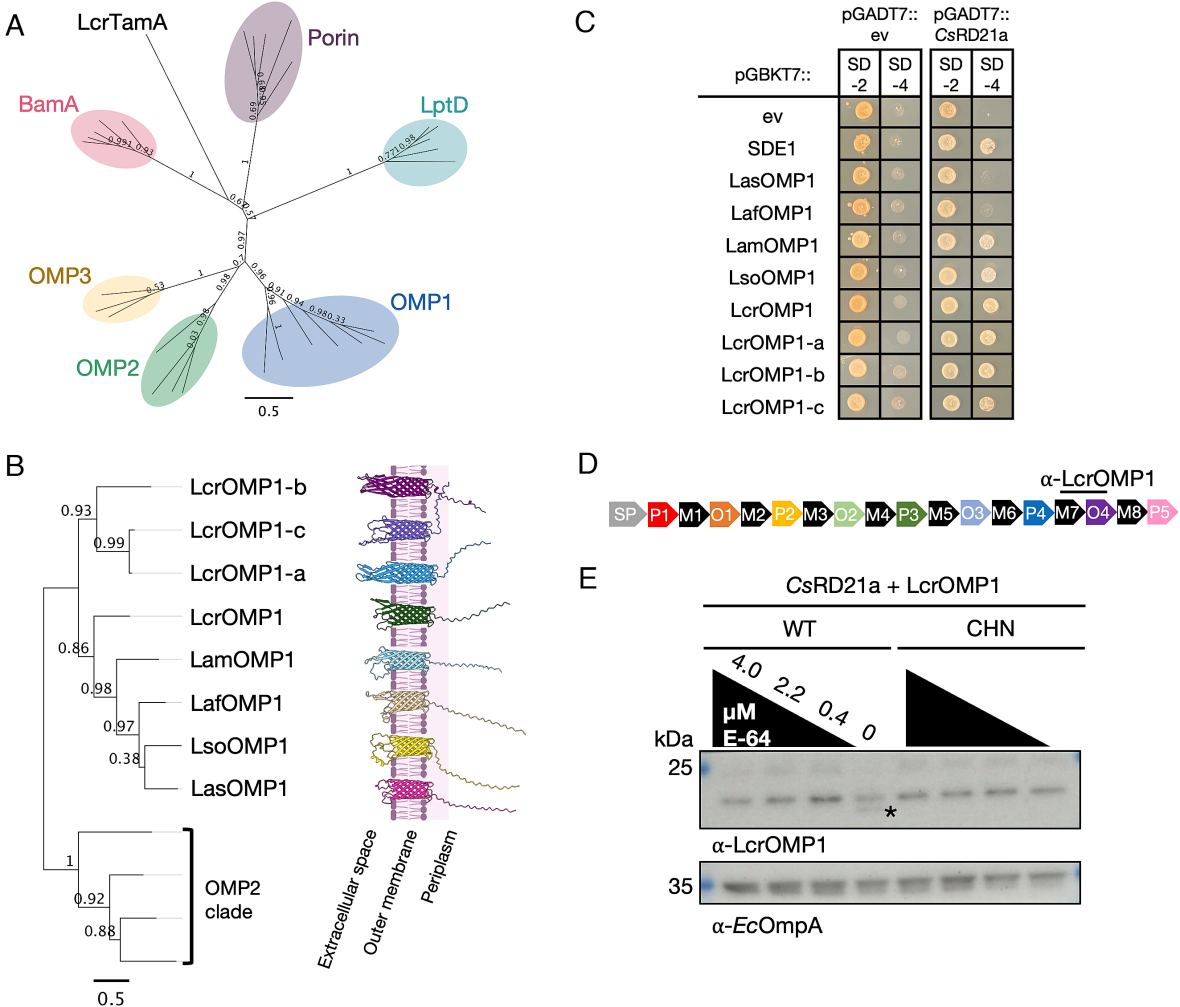
*Cs*RD21a interacts with OMP1 homologs in *Liberibacter* spp. and weakly cleaves LcrOMP1. (*A*) Unrooted maximum likelihood tree depicting six clusters of 31 predicted OMPs in Liberibacter (Dataset S1). A MAFFT amino acid sequence alignment was used to generate the phylogeny. FastTree support values are indicated at each node. Tree is visualized using Geneious Prime. (*B*) Phylogenetic tree produced from a MAFFT alignment of Liberibacter OMP1 proteins, with the OMP2 cluster as an outgroup. AlphaFold3 structural models of the OMP1 proteins are shown beside the tree. All pTM scores for the models were between 0.65 and 0.73, and the membrane image was sourced from BioRender.com. (*C*) Yeast two-hybrid assays detecting interactions between OMP1 homologs with *Cs*RD21a. One colony was diluted to OD_600_ = 0.4 and plated for each cotransformation in yeast on both SD-2 and SD-4 media. All proteins were cloned without their signal peptides. (*D*) Domain structure of LcrOMP1 depicting the binding site of the α-LcrOMP1 antibody. (*E*) Semi-in vitro cleavage assay of LcrOMP1 by *Cs*RD21a. Wild-type (WT) and catalytic mutant (CHN) *Cs*RD21a were incubated with *E. coli* membrane fractions containing LcrOMP1. Cleaved product of LcrOMP1 is marked with the asterisk. *Ec*OmpA was used as a loading control.

To further understand LasOMP1 as a putative target of plant defense, we identified its homologs in other Liberibacter species, which include the citrus pathogens Laf and Lam, the potato pathogen Lso, and a nonpathogenic, culturable relative, *L. crescens* (Lcr) (Dataset S1). Phylogenetic analysis of 31 HMM-predicted Liberibacter OMPs revealed conserved clades corresponding to LptD, Porin, and BamA, which each contain one protein from each species (Fig. 3*A*). Interestingly, we observed a conserved and expanded cluster comprising LasOMP1 and a single representative homolog from each Liberibacter proteome, apart from Lcr which has four homologs (Fig. 3 *A* and *B*). In contrast, OMP2 and OMP3 are specific to pathogenic “*Ca.* Liberibacter” spp., suggesting they may have a role in pathogenesis.

In Las, *OMP1* is one of the most highly expressed genes in both the plant host and the insect vector (*SI* *Appendix*, Fig. S1*B*). To determine if OMP1 is also the dominant OMP in Lcr, we performed RNAseq analyses of this bacterium grown in liquid medium. This analysis revealed that LcrOMP1 homologs are also the highest expressed OMPs and each LcrOMP1 paralog was among the top 17% most abundant Lcr transcripts (*SI* *Appendix*, Fig. S3). The LcrOMP1 homologs form a similar AlphaFold-predicted protein model in which the transmembrane *β*-barrel and periplasmic disordered region are structurally conserved (Fig. 3*B*). However, their extracellular loops appear to have diverged and, in some cases, expanded. Consistent with this prediction, sequence alignment of amino acid sequences shows a lower degree of conservation in the O domains as compared to M domains, indicating potential functional diversification (*SI* *Appendix*, Fig. S4).

To determine the extent to which OMP1 is conserved across Gram-negative bacteria, we expanded the prediction of OMPs to include 20 proteomes including representative plant pathogens, symbionts, and animal pathogens (*SI* *Appendix*, Fig. S5*A*). A total of 964 OMPs were predicted and further categorized based on the number of transmembrane domains (*SI* *Appendix*, Fig. S5*B* and Dataset S2). We then extracted 8-stranded *β*-barrel OMPs and generated a phylogenetic tree. We found that OMP1 forms a distinct clade with several OMPs from *Agrobacterium tumefaciens* and *Sinorhizobium meliloti*, which belong to the Rhizobiaceae family together with Liberibacter (*SI* *Appendix*, Fig. S5*C*). In contrast, the other 8-stranded Liberibacter OMPs, OMP2, and OMP3, are unique to Liberibacter. Further, there is a sister lineage of OMP1 that includes OMPs from two *Xanthomonas* species*, X. citri* and *X. campestris*, both of which are plant pathogens. These results suggest that OMP1-like proteins are encoded in a variety of plant-associated bacteria and potentially engaged in host interactions.

The expression pattern and conservation of OMP1 proteins suggests that they may play an important role in bacterial growth and thus could serve as targets of plant defense. These observations prompted us to further study this family of proteins as putative *Cs*RD21a substrates. We found that *Cs*RD21a interacts with each tested OMP1 homolog using yeast-two-hybrid assays (Fig. 3*C*). Both LasOMP1 and LafOMP1 exhibit weak interactions with *Cs*RD21a, as evidenced by less yeast growth on the selective media, compared to the other OMP1 proteins, which may indicate protease–substrate interactions.

To assess whether *Cs*RD21a cleaves the OMP1 homologs, we systematically tested them using the semi-in vitro assay. Because adding a C-terminal tag to OMPs for expression in *E. coli* obscured the identification of mature proteins from the membrane extracts, we generated an antibody, α-LcrOMP1, based on the M7-O4 region of LcrOMP1 that would allow us to monitor potential cleavage of LcrOMP1 proteins (Fig. 3*D*). Incubation of AF containing wild-type *Cs*RD21a but not *Cs*RD21a^CHN^ with LcrOMP1 resulted in the appearance of a cleaved product only in the absence of the protease inhibitor E-64 (Fig. 3*E*). This is in stark difference with what we observed in LasOMP1, which was efficiently cleaved by *Cs*RD21a even in the presence of E-64 at a concentration as high as 2.2 *µ*M. These results suggest that OMP1 proteins may be common targets of plant PLCPs, but the strength of *Cs*RD21a interaction, and the efficacy of cleavage, varies for different OMP1 homologs.

### *Cs*RD21a Cleaves LasOMP1 at its N-terminus.

**Fig. 4.**
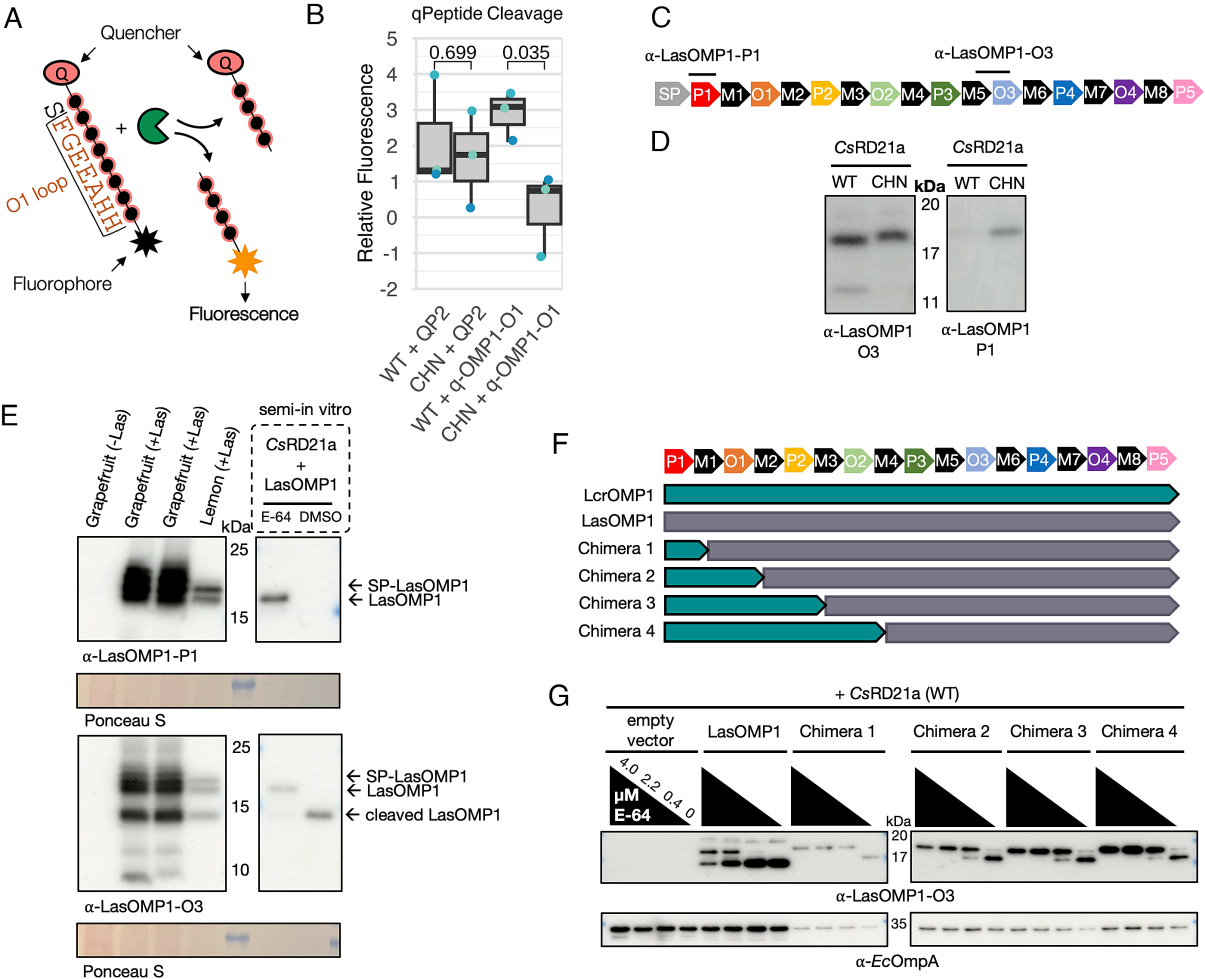
*Cs*RD21a cleaves within the N-terminal region of LasOMP1. (*A*) Experimental design using a quenched octopeptide (qPeptide) to detect protease-based on cleavage. (*B*) *Cs*RD21a cleaves the O1 loop of LasOMP1. Apoplastic fluid containing wild-type (WT) or the catalytic mutant (CHN) of *Cs*RD21a was extracted from *N. benthamiana* and incubated with qPeptides corresponding to flg22 (QP2) or the O1 loop of LasOMP1 (q-OMP1-O1). Relative fluorescence unit (RFU) values from inhibited (E-64-treated) apoplastic fluid were subtracted from uninhibited (DMSO-treated) for each protease-qPeptide combination after 30 min, and the resulting values were normalized to the WT and CHN controls, respectively, without the qPeptides. Data from three independent biological replicates were presented (corresponding to the different colored data points), and the p-values shown between WT and CHN treatments were derived from two-tailed *t* tests. (*C*) Schematic depicting the domain structure of LasOMP1 and the binding site of the α-LasOMP1-P1 antibody. (*D*) Western blots showing the different *Cs*RD21a wild-type (WT)- and catalytic mutant (CHN)- produced cleavage products detected by different LasOMP1-specific antibodies. (*E*) Western blotting of healthy (-Las) and infected (+Las) citrus seed coat vasculatures detecting LasOMP1. Samples were run alongside *Cs*RD21a-cleaved LasOMP1 from the semi-in vitro assay. Infected grapefruit samples are duplicates. Ponceau S was used as the loading control. (*F*) Schematic depicting the LcrOMP1 and LasOMP1 domain structure and the chimeras used in semi-in vitro cleavage assays shown in panel G. (*G*) Western blots showing the cleavage of OMP1 chimeras by *Cs*RD21a detected by the *α*-LasOMP1-O3 antibody. *Ec*OmpA was used as a loading control.

Our yeast-two-hybrid results suggested that the N-terminal region of LasOMP1 mediates interaction with *Cs*RD21a. This region includes the O1 loop, which is predicted to be exposed to the cell surface of Las. Furthermore, cleavage in this region would produce a ~14 kDa product, which is similar to what was detected using the α-LasOMP1-O3 antibody. Therefore, we hypothesized that the O1 loop is cleaved by *Cs*RD21a. To test this, we synthesized a quenched octopeptide including one amino acid of the M1 domain and the following seven amino acids of the O1 loop of LasOMP1 (q-OMP1-O1) (Fig. 4*A*). Upon cleavage of the peptide, the N-terminal quencher would be separated from the C-terminal fluorophore, causing fluorescence (16, 17). We found that incubation of the wild-type *Cs*RD21a-containing AF with q-OMP1-O1 resulted in a significantly higher level of fluorescence compared to the *Cs*RD21a^CHN^ mutant (Fig. 4*B*). As a control, a quenched octopeptide derived from the bacterial flagellin flg22 (QP2) that was previously found to be processed by subtilases but not PLCPs (16) did not emit fluorescence that was different between wild-type *Cs*RD21a and *Cs*RD21a^CHN^ treatments. This result suggests that the O1 loop of LasOMP1 can be cleaved by *Cs*RD21a. We also examined the OMP1 homolog in the Las strain Ishi-1, which contains a single nucleotide polymorphism, resulting in a histidine to aspartic acid mutation at the 73^rd^ amino acid in the O1 loop (*SI* *Appendix*, Fig. S6*A*). LasOMP1^Ishi-1^ could also be cleaved by *Cs*RD21a (*SI* *Appendix*, Fig. S6*B*), suggesting that *Cs*RD21a is effective in targeting naturally occurring variants of OMP1 in Las.

We further characterized the cleavage of LasOMP1 by *Cs*RD21a using Western blotting. For this purpose, we generated an antibody against the P1 domain of LasOMP1, which should be in the proximity of the potential cleavage site (Fig. 4*C*). Notably, we were unable to detect any *Cs*RD21a-produced cleavage products of LasOMP1 using the α-LasOMP1-P1 antibody, although the cleavage products were clearly detectable using the α-LasOMP1-O3 antibody (Fig. 4*D*). Nonetheless, full-length LasOMP1 proteins were largely reduced in Western blots using the α-LasOMP1-P1 antibody in the presence of wild-type *Cs*RD21a, suggesting that the cleavage likely occurs in the proximity of the antibody-binding site.

We next tested whether LasOMP1 is cleaved in citrus during Las infection using the α-LasOMP1-O3 and α-LasOMP1-P1 antibodies by Western blotting. Total proteins were extracted from HLB-infected citrus seed coat vasculatures, which had high populations of Las (42, 43). Two bands larger than 15 kDa appeared using both antibodies, likely corresponding to the SP-containing, nonsecreted LasOMP1 protein (21.5 kDa) and the SP-lacking, secreted LasOMP1 protein (19.2 kDa), respectively (Fig. 4*E*). The 19.2 kDa band is the same size as the membrane-incorporated LasOMP1 proteins produced in *E. coli*. Interestingly, an additional <15 kDa band, likely representing the cleavage product by *Cs*RD21a, was detectable using the α-LasOMP1-O3 antibody but not the α-LasOMP1-P1 antibody, similar to what was observed in the semi-in vitro assay (Fig. 4*E*). No LasOMP1 signal was detectable in healthy citrus samples using these antibodies, confirming their specificity. Together, these results suggest that *Cs*RD21a cleaves LasOMP1 *in planta* during natural infection.

The relatively weaker cleavage of LcrOMP1 than LasOMP1 by *Cs*RD21a allowed us to further dissect the cleavage site of LasOMP1 by generating chimeras. We generated four chimeras that contained increasingly larger N-terminal fragments of LcrOMP1 and shorter fragments of the C-terminus of LasOMP1 while retaining the binding site of the α-LasOMP1-O3 antibody (Fig. 4*F*). These chimeras were individually expressed in *E. coli* and subjected to the semi-in vitro cleavage assay. Intriguingly, all the chimeras exhibited reduced cleavage efficiency compared to LasOMP1 (Fig. 4*G*), indicating the N-terminal region of LcrOMP1, containing only the P1 domain and two amino acids of the adjacent M domain, is sufficient to reduce the cleavage efficiency by *Cs*RD21a. Together with the q-OMP1-O1 cleavage assay, our results suggest that LasOMP1 may cleave multiple sites in the N-terminal region, which is consistent with our previous finding that the P1 and O1 domains both mediate *Cs*RD21a interaction.

### *Cs*RD21a Overexpression Enhances Las Resistance in Citrus.

**Fig. 5.**
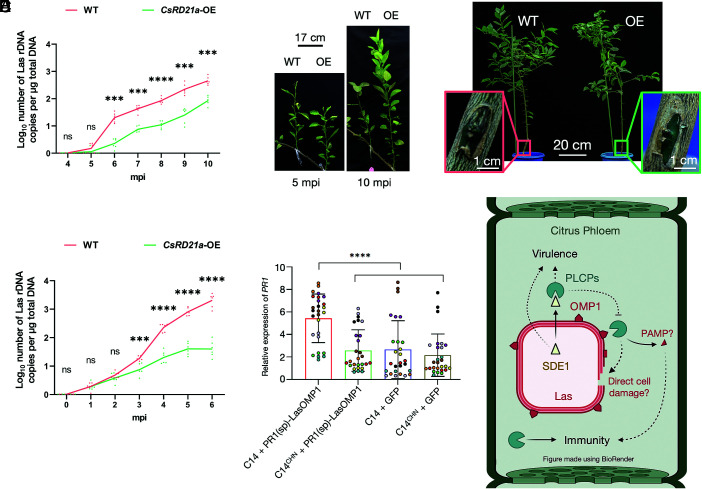
RD21a reduces Las growth in citrus and activates a LasOMP1-dependent immune response. (*A*) qPCR determination of Las populations in wild-type (WT) and transgenic sweet orange scions overexpressing *CsRD21a* (OE), which were grafted on Las-infected citrus plants. Data points represent six independent biological replicates. Asterisks indicate significant differences based on Student’s *t*test: ns = no significance and **** = *p* < 0.0001. Las population was determined at 4 to 10 mo post inoculation (mpi) as Las rDNA copy numbers per *µ*g of DNA. (*B*) Images of grafted WT or *CsRD21a*-OE scions on HLB-infected citrus from two representative time points. Additional images can be found in *SI* *Appendix*, Fig. S8. (*C*) Images of WT and *CsRD21a*-OE sweet orange rootstocks graft-inoculated with Las-infected buds at 9 mo post inoculation. Pink and green boxes show the sites of graft-inoculation on WT and *CsRD21a*-OE plants, respectively. (*D*) qPCR determination of Las populations in WT and *CsRD21a*-OE sweet orange rootstock plants graft-inoculated with Las-infected buds. Data points represent six independent biological replicates. Asterisks indicate significant differences based on Student’s *t* test: ns = no significance, ***P < 0.001, and ****P < 0.0001. (*E*) Relative expression of *PR1* determined by RT-qPCR in *N. benthamiana* leaves transiently coexpressing C14 (WT or CHN) and PR1(sp)-LasOMP1 or GFP. Nine independent biological replicates (shown by different colored data points) were analyzed. Three technical replicates were included for each biological replicate. Relative expression levels were calculated using the 2^−ΔΔCT^ method with *Actin* as the internal control. Data are presented as the mean ± standard deviation. Statistical significance was determined by one-way ANOVA, where ****P < 0.0001. (*F*) Model of PLCP-dependent defense in the citrus during Las infection. PLCPs, such as *Cs*RD21a, contribute to HLB resistance by targeting pathogen-derived substrates, such as LasOMP1. Cleavage of OMP1 may directly induce bacterial cell damage or lead to immune activation, potentially through the production of peptide(s) with PAMP activities. As a counterdefense, Las secretes an effector, SDE1, which acts as a protease inhibitor to block PLCP-dependent defense and contribute to pathogen virulence. Created in BioRender. Lab, W.M. (2026) https://BioRender.com/b2cxdwh.

Upon HLB infection, proteases are dynamically regulated in citrus (26). PLCP expression is induced in the tolerant variety Sugar Belle mandarin but decreased in the susceptible variety Pineapple sweet orange (27), suggesting that PLCPs may contribute to HLB resistance. We overexpressed *CsRD21a* without a tag (given its proteolytic processing in planta) under the 35S promoter in sweet orange. Increased transcript abundance of *CsRD21a* in the transgenic plants compared to wild-type plants was confirmed by RT-qPCR (*SI* *Appendix*, Fig. S7*A*). Six independent transgenic lines with ~20-fold higher expression of *CsRD21a* were examined in Las infection experiments. No visible differences in growth and development were observed between *CsRD21a*-overexpressing (*CsRD21a-*OE) and wild-type (WT) citrus plants under normal growth conditions. We then examined Las infection of *CsRD21a*-OE and WT plants by grafting transgenic buds onto the same branches of HLB-infected sweet orange plants (*SI* *Appendix*, Fig. S7*B*). Las populations were monitored monthly by qPCR in the scions, and a standard curve was generated to correlate Ct values to copy numbers of 16S rDNA. Las rDNA was confidently detected at 6 mo postinoculation (mpi). From 6 mpi until the end of the 10-mo monitoring period, Las populations were significantly lower in *CsRD21a*-OE than WT scions with ~10-fold less Las rDNA in the *CsRD21a*-OE scions (Fig. 5*A*). Additionally, *CsRD21a*-OE scions grew taller with more overall leaf area than WT scions during this time (Fig. 5*B* and *SI* *Appendix*, S8), suggesting that *CsRD21a* overexpression counteracted the negative impacts of Las infection on citrus canopy growth that is commonly observed in HLB-diseased citrus (19).

To further confirm the role of *CsRD21a* in defense during Las infection, we performed the reciprocal experiment in which *CsRD21a*-OE and WT citrus plants were graft-inoculated with Las-infected buds (Fig. 5*C*). Las growth was monitored over a 6-mo period via qPCR. From 3 mpi, *CsRD21a-*OE lines contained significantly less Las rDNA than the WT with an ~100-fold reduction at the end of the 6-mo monitoring period (Fig. 5*D*). Taken together, these results demonstrate that overexpression of *CsRD21a* reduced Las proliferation in citrus using two different Las inoculation assays.

### LasOMP1 Activates Defense in *N. benthamiana*.

Cleavage of LasOMP1 may contribute to defense through multiple mechanisms. Given the high expression level of *LasOMP1*, it may have essential functions, thus its cleavage likely inhibits Las growth. Unfortunately, this cannot be directly tested since Las is unculturable. Furthermore, LasOMP1 cleavage may release peptide(s) that activates plant immunity. To test this, we coexpressed LasOMP1 in *N. benthamiana* with *Cs*RD21a or its tomato homolog C14, retaining the SPs of both PLCPs. To ensure secretion of LasOMP1 into the apoplast, we replaced its native SP with the SP of pathogenesis-related protein 1 (PR1). In this experiment, we consistently observed more efficient LasOMP1 cleavage by C14 than *Cs*RD21a (*SI* *Appendix*, Fig. S9*A*). Indeed, C14, but not C14^CHN^, is highly active in *N. benthamiana* apoplastic fluid and cleaves LasOMP1 in the semi-in vitro assay (*SI* *Appendix*, Fig. S9 *B* and *C*). Therefore, we used C14 to test for potential immune activation upon LasOMP1 cleavage, using *PR1* expression as a proxy. C14 or C14^CHN^ did not induce *PR1* expression when coexpressed with GFP, indicating that its proteolytic activity alone could not activate immunity in *N. benthamiana* (Fig. 5*E* and *SI* *Appendix*, Fig. S9*D*). In contrast, wild-type C14 coexpressed with LasOMP1 significantly induced *PR1* expression compared to C14^CHN^, suggesting that LasOMP1 activates immunity only when proteolytically processed.

We next sought to test whether a putative RD21a-produced N-terminal peptide of LasOMP1 is sufficient for *PR1* induction. For this purpose, we synthesized a 51-amino acid peptide corresponding to the P1-M1-O1 region of LasOMP1 (*SI* *Appendix*, Fig. S10*A*) and infiltrated the purified peptide directly into *N. benthamiana* leaves. However, unlike flg22, the P1-M1-O1 peptide did not induce *PR1* expression at 6 h postinfiltration (*SI* *Appendix*, Fig. S10*B*). Therefore, we speculate that the activation of defense upon RD21a cleavage of LasOMP1 is triggered by a yet undefined peptide in the larger C-terminal cleavage product.

Together, our data clearly demonstrate that *Cs*RD21a overexpression contributes to HLB resistance and that LasOMP1 is cleaved by *Cs*RD21a. Due to the inherent technical challenges of the Las-citrus pathosystem, a direct link between the cleavage of LasOMP1 and the defense activity of *Cs*RD21a against HLB has yet to be established based on the current evidence. Demonstrating a direct effect of LasOMP1 cleavage on bacterial growth is not feasible as Las is not cultivable. In addition, monitoring the release of cleaved LasOMP1 peptide(s) in infected citrus trees is a challenging endeavor due to the overall low Las titer. Nonetheless, by demonstrating that LasOMP1 activates the expression of a defense marker gene in an RD21a-dependent manner via heterologous expression in *N. benthamiana*, we propose that RD21a-dependent cleavage of LasOMP1 may directly damage and kill Las cells and/or activate an immune response (Fig. 5*F*). Future research is required to further support this model.

## Discussion

The class of RD21-like PLCPs has been implicated in plant immunity through genetic studies and the identification of numerous pathogen-derived inhibitors; yet direct targeting of pathogen-derived substrates by any plant PLCP as a defense strategy has not been demonstrated. Here, we identified bacterial OMPs as direct targets of plant PLCPs. Specifically, we observed that OMP1 from the citrus HLB pathogen Las is specifically cleaved by the citrus RD21a. LasOMP1 is one of the most highly expressed Las genes and is significantly more highly expressed than other Las OMPs. Similarly, our RNAseq analyses on the culturable relative of Las, Lcr, also revealed high expression of LcrOMP1, suggesting that this family of OMPs may have essential functions in Liberibacter growth, making them attractive targets of plant defense mechanisms.

Previously, antibody-based HLB detection has been developed by targeting a Las “OMP” or “OmpA” (44, 45). However, we found that these are the same protein as LasBamA. Peptides derived from another gene previously identified as Las OmpA (CLIBASIA_04260) have been found to reduce Las acquisition by its insect vector (46). However, according to DeepTMHMM prediction, the CLIBASIA_04260 protein lacks a transmembrane domain; therefore, it was not identified as an OMP in our analysis. We hope our systematic analyses of *β*-barrel OMPs can clarify the nomenclature (Dataset S1).

Our results suggest that the LasOMP1 N-terminal region, including the extracellular O1 loop and the periplasmic P1 domain, mediates both the interaction and cleavage by *Cs*RD21a. It is possible that the O1 loop interacts with *Cs*RD21a in the extracellular fluid in planta and is cleaved by this host enzyme as a defense response. This cleavage may disrupt the *β*-barrel structure of LasOMP1 and expose the periplasmic P1 domain, which can be cleaved by *Cs*RD21a subsequently. Future work should further characterize the cleavage site(s) and clarify how LasOMP1-derived peptide(s) may be released from *Cs*RD21a-dependent cleavage to activate immunity. Importantly, very little is known about how defense is mounted in the phloem, posing a major limitation in this field of study. The identification of OMP1 as a target of host proteases opens avenues to elucidate the fundamental principles of phloem-based immunity. OMP1-like proteins are also encoded in apoplast-residing pathogens, such as *A. tumefaciens* and *Xanthomonas spp,* indicating that they may play a broader role in plant–pathogen interactions. These culturable bacteria may represent suitable systems to further elucidate the function of OMP1 homologs in bacterial growth, host colonization, and PLCP-mediated plant defense in future studies.

OMPs are abundant, cell surface-exposed proteins that contribute to membrane integrity, virulence, and nutrient uptake in Gram-negative bacteria and have been shown to be immunogenic in animal systems (29, 47, 48). In *E. coli*, it is estimated that OMPs cover over half of the OM surface. For example, there are >100,000 copies of *Ec*OmpA alone in the OM (28). A human serine protease, called neutrophil elastase (NE), cleaves *Ec*OmpA, resulting in bacterial membrane instability and growth inhibition (49, 50). As such, the serine protease has direct antimicrobial activities. In this study, LasOMP1 cleavage may directly affect Las growth via reducing the integrity of the bacterial membrane and/or indirectly inducing plant immunity by facilitating the release of PAMPs that are normally not exposed to the host. Our data also suggest that a PAMP may be derived from LasOMP1 cleavage. Therefore, animal and plant proteolytic enzymes may target bacterial pathogen OMPs as a conserved strategy but activate defense through different mechanisms.

Similar to previous findings that OMPs in animal pathogens undergo extracellular loop diversification (51), the OMP1 homologs also exhibit sequence diversification at the extracellular loop region. Future research should systematically investigate the impact of this diversification on the functions and defense activation of OMP1. Intriguingly, LcrOMP1 has been found via proteomic analyses in the outer membrane vesicles (OMVs) of Lcr (52). *Ec*OmpA is also OMV-associated, and its exogenous application to *E. coli* cells has been shown to reduce NE-mediated killing (53), potentially by sequestering the antimicrobial proteases away from the bacterial cell surface to protect cell integrity. It is compelling to speculate that Liberibacter may also use a similar strategy to compensate PLCP activities by highly expressing OMP1.

Our data show that overexpression of *Cs*RD21a in citrus enhances HLB resistance by reducing Las populations by 10 to 100 folds. Las can cause disease with low titers and patchy distribution in the phloem (54). Therefore, this level of reduction in Las bacterial load may significantly reduce HLB disease progression in the long term. We found that a naturally occurring mutant of LasOMP1, LasOMP1^Ishi-1^, is also cleaved by *Cs*RD21a, suggesting that *Cs*RD21a engineering in citrus may be effective against various Las strains. PLCPs are widespread in plants, representing nine major families (55), thus may contribute to defense against a wide variety of pathogens. For example, C14 may be an exciting target for engineering resistance to Lso in tomato and potato. Yet, unlike *Cs*RD21a, C14 also cleaves *Ec*OmpA in the semi-in vitro cleavage assay, suggesting there is more to be uncovered regarding PLCP specificity against OMPs. Taken together, our results identified a pathogen substrate of secreted proteases in plant defense, shedding light on the convergence of plant and animal immune systems on targeting bacterial OMPs and offering opportunities to engineering defense against the devastating HLB pathogen.

## Materials and Methods

### OMP Prediction and Analyses.

For OMP prediction, bacterial proteomes were downloaded from NCBI (https://www.ncbi.nlm.nih.gov/). PFAM alignments for the membrane *β*-barrel clan (MBB-CL0193) were downloaded, and hmmbuild was used to build the HMM (30). Next, hmmsearch (https://hmmer.org/) was used to scan each proteome for the presence of OMPs using the HMM. The resulting OMPs were screened using DeepTMHMM (31) and only proteins identified by both programs were subjected to further analyses. The number of transmembrane domains predicted by DeepTMHMM were used to categorize each OMP for subsequent phylogenetic analyses. MUSCLE v5.1 (56) or MAFFT v7.490 (57) alignments were generated, as indicated, of FASTA amino acid sequences. Subsequent maximum likelihood model FastTree v2.1.11-generated phylogenies (58, 59) were made and visualized using Geneious Prime (https://www.geneious.com/) and ggtree (60). A complete list of the OMPs predicted for this study is in Dataset S2.

### Yeast-Two-Hybrid.

The yeast strain AH109 (Takara) was maintained in YPDA (Takara) medium prior to transformation. Overnight AH109 liquid cultures were subcultured until an OD_600_ between 0.5 and 0.8 was reached. The cells were then washed twice in sterile water followed by pelleting at 1,000×*g* for 5 min prior to resuspension in transformation buffer, which comprised 50% polyethylene glycol (PEG) 3350, 1M lithium acetate pH 7.0, and 10× Tris-EDTA buffer pH 8.0, at a ratio of 8:1:1. Salmon sperm DNA (Invitrogen) was then added to the transformation mixture with ~2,000 ng of both bait and prey vectors (Takara), and the cells were incubated at 42 °C for 45 min. The cells were then pelleted and resuspended in 0.9% NaCl before being plated on YPAD medium. Cotransformed colonies were serially diluted in sterile water after normalizing the OD_600_ as indicated for each experiment and replated on SD/-Trp/-Leu (SD-2) and SD/-Trp/-Leu/-His/-Ade (SD-4) (Takara). Primers for pGBKT7 cloning can be found in Dataset S3. LcrOMP1a/b/c, LsoOMP1, LamOMP1, and LafOMP1 genes were synthesized as gene fragments (Twist). *Cs*RD21a and *Cs*SAG12-1 in the pGADT7 backbone and LasSDE1 in the pGBKT7 backbone were sourced from Clark et al. (24). LasOMP1 in the pGBKT7 backbone was sourced from Dr. Simon Schwizer.

### Apoplastic Fluid Extraction.

*Cs*RD21a and C14 (as well as their catalytic CHN mutants) were cloned into the binary vector pJK268c (provided by Dr. Jiorgos Kourelis) which contains the RNA silencing suppressor P19 in the backbone (61). Expression was driven by a 2x35S CaMV promoter. *Cs*RD21a and C14 were cloned in their full-length forms without a tag into pJK268c via the pICH41308 Golden Gate level 0 acceptor. Primers can be found in Dataset S3. These constructs were transformed into *A. tumefaciens* GV3101 competent cells and infiltrated in the leaves of 4 to 6-wk-old *N. benthamiana* plants, which were grown in controlled conditions at 25 °C with a 16-h light/8-h dark cycle. At 3 d postinoculation, the leaves were removed, vacuum infiltrated with sterile water, and centrifuged at 1,500 rpm at room temperature for 25 min in the barrel of a 20 mL syringe inserted in a 50 mL Eppendorf tube. The flow-through apoplastic fluid from these leaves was then concentrated 2X using 10 kDa concentrator columns (Thermo) and subjected directly to activity-based protein profiling or the semi-in vitro OMP cleavage assay.

### Activity-Based Protein Profiling (ABPP).

Concentrated apoplastic fluid was collected as mentioned above and was incubated for 30 min at room temperature with concentration gradients of E-64 (diluted with DMSO) or just DMSO at room temperature. DCG-04 (MedKoo Biosciences, Inc.) was then added to a final concentration of 500 *µ*M with 5 mM dithiothreitol (DTT) and 50 mM sodium acetate pH 5.0. After 1 h incubation at room temperature, 3x protein loading dye (30% glycerol, 3% SDS, 0.1 mM Tris HCl pH 6.8, 0.05% bromophenol blue) was added, and the samples were boiled prior to SDS-PAGE analysis. Protein samples were transferred from the gel to a PVDF membrane (Bio-Rad), and the membrane was subsequently blocked in 3% BSA then incubated in a 1:10,000 dilution of Streptavidin-HRP (Thermo) in 3% BSA. Active proteases labeled by DCG-04/Streptavidin-HRP were detected via chemiluminescence imaging.

### *E. coli* Membrane Enrichment of OMPs.

Tag-free, outer membrane proteins without their signal peptides were N-terminally fused to the *E. coli* OmpA signal peptide, synthesized as gene fragments (Twist), and cloned into the pOPIN-F6-C vector under the control of the T7 promoter (62). Primers can be found in Dataset S3. These constructs were transformed into *E. coli* C41 competent cells (63) and grown in liquid autoinduction media at 30 °C until OD_600_ of 0.5 to 0.7 was reached, at which point the cultures were moved to 18 °C overnight. Pellets were collected via centrifugation at 5,500 rpm (6,561×*g*) at 4 °C and were resuspended in A1 buffer (200 mM Tris, 200 mM glycine, 20% glycerol, 2M NaCl, and 80 mM imidazole) with cOMPLETE Protease Inhibitor Cocktail tablets (Sigma). The cells were sonicated and centrifuged again at 5,000 rpm (2,990×*g*) and 4 °C for 30 min. The supernatant was then loaded into PA Ultracrimp tubes (Thermo) and ultracentrifuged at 27,500 rpm (77,077×*g*) for 10 min. The waxy pellet was then resuspended in A1 buffer with 1% DDM (n-dodecyl-*β*-D-maltoside) and incubated with rotation at 4 °C overnight.

### Semi-in vitro Protease Cleavage Assay.

*E. coli* membrane fractions containing OMPs were directly incubated with concentrated apoplastic fluid for 3 h at room temperature with final concentrations of 5 mM DTT and 50 mM sodium acetate pH 5.0. As in the ABPP method, 3x protein loading dye was added to the samples, which were then boiled. These samples were then run on SDS-PAGE gels and analyzed via Western blotting using custom monospecific OMP1 antibodies raised in rabbits (Pacific Immunology). LasOMP1-specific antibody “α-LasOMP1-O3” was raised against the synthetic peptide GPDVAQKYETGKAGEIT, LasOMP1-specific antibody “α-LasOMP1-P1” was raised against the synthetic peptide DPVRRAHHGGRGVVPTIATN, and LcrOMP1-specific antibody “α-LcrOMP1” was raised against the synthetic peptide RLEYRYTRLGKKDFTLRDA. As a control, *Ec*OmpA was detected using α-*Ec*OmpA (Antibody Research Corporation). All blocking was performed using 3% milk. The primary antibodies were diluted to 1:2,500. Secondary anti-rabbit antibody (Invitrogen) was diluted to 1:10,000.

### Quenched Peptide Cleavage Assay.

Apoplastic fluid containing wild-type or CHN mutant *Cs*RD21a was collected as above and incubated with 4.0 *µ*M E-64 or DMSO for 30 min. 5 mM dithiothreitol (DTT) and 50 mM sodium acetate pH 5.0 was then added to 1 *µ*M synthetic peptides QP2 (LKINSAKD) or q-OMP1-O1 (SFGEEAHH). Each peptide was synthesized with an N-terminal DABCYL quencher and a C-terminal Glu-EDANS fluorophore. Fluorescence was immediately measured every minute for 30 min at room temperature using a SpectraMax ID5 plate reader with a 335 nm excitation wavelength and a 493 nm emission wavelength. QP2 was sourced from Buscaill*, et al.* (16), and q-OMP1-O1 was generated for this study (Genscript).

### OMP1 Detection in Healthy and Infected Citrus.

Fruits from infected grapefruit and lemon trees were collected from groves at UF CREC in Lake Alfred, FL. The seeds were isolated from the fruits, and the seed coats were carefully removed with forceps. The seed vasculatures were then collected with forceps, pooled together, and frozen in liquid nitrogen. These samples were then ground with a mortar and pestle and put on ice. After adding protein loading dye to the samples, they were boiled for 10 min and centrifuged at 16,100×*g* for 5 min. The supernatant was then subjected to SDS-PAGE and subsequent Western blot analyses using the LasOMP1-specific antibodies as above.

### RNAseq Analysis of *L. crescens*.

Duplicate 3 mL *Liberibacter crescens* BT-1 cultures were grown in liquid BM7 media for 7 d shaking at 150 rpm at 28 °C (64, 65). Cultures were spun down at 15,000×*g* for 5 min at 4 °C, and RNA was extracted from bacterial pellets using a Trizol-based extraction protocol (33). The RNA extracts were then treated with DNase I (Thermo Fisher Scientific). The RNA concentration and the quality of the extracts were determined using a NanoDrop spectrophotometer and an Agilent 2100 Bioanalyzer with prokaryote analysis software. RNA libraries were constructed with a TruSeq Stranded Total RNA Library Prep Kit (Illumina). The RNA samples were treated with the Illumina Ribo-Zero Plus rRNA Depletion Kit as part of the standard protocol. cDNA libraries were then subjected to RNA-seq with an Illumina HiSeq system with 150-bp strand-specific paired end reads. Library preparation and sequencing were performed by Genewiz.

The quality of the raw sequencing data was checked with FastQC v.0.11.9 (66). To ensure high-quality sequences for mapping and downstream analyses, low-quality reads and an adapter were trimmed using Trimmomatic v.0.39 (67). RNA-seq reads were aligned with the indexed Lcr BT-1 genome assembly (NC_019907.1) and were parsed to it using HISAT2 v.2.2.1 (68). The number of reads mapped to each gene was counted using the feature counts function of the Rsubread package (69). The data used for the analysis have been deposited into the European Nucleotide Archive database (BioProject PRJEB82095). Read counts were normalized across all samples using the counts function in DESeq2 package v.1.30.1 from Bioconductor (70). Data can be found in Dataset S4.

### Generation of Transgenic Citrus.

To generate constructs for plant transformation, the full-length coding sequence of *CsRD21a* was amplified from sweet orange with gene-specific primers and cloned into the pDONR221 vector and then fused into the binary vector pK7WG2D using BP and LR enzymes. The *A. tumefaciens* strain EHA105 carrying the recombinant plasmid was used for sweet orange transformation as described previously (71). *CsRD21a*-transgenic citrus plants were confirmed using RT-qPCR with gene-specific primers. Total RNA was extracted from transgenic and WT citrus using Trizol iso plus (Takara). Total RNA was reverse transcribed to cDNA using the HiScript II Reverse Transcriptase Kit (Vazyme Biotech). The RT-qPCR was performed with Hieff® qPCR SYBR Green Master Mix (YEASEN) and Light Cycler 480 (Roche). With the citrus *β*-Actin gene as the internal reference gene, the data were normalized using the 2^−∆∆Ct^ method. All primers used are listed in Dataset S3. All the transgenic and control plants were grown in a greenhouse at 25 to 30 ˚C.

### Leaf Area Measurement.

The total leaf area was measured using ImageJ (imagej.net). All leaf images were converted to 8-bit grayscale, and a spatial scale was set based on the scale bar in each image to convert pixel units into physical units (mm); this scale was then applied to all images. Grayscale images were subsequently converted to binary images using an automatic thresholding method to segment leaf areas. When leaf boundaries were unclear, manual threshold adjustments were applied to improve segmentation accuracy. Leaf areas were then analyzed using the Analyze Particles function, with a minimum area threshold set to exclude background noise and small artifacts, thereby obtaining total leaf area. For samples with low contrast between leaves and the background, leaf areas were manually delineated using the freehand selection tool and measured directly. The measurements were performed using three independent transgenic lines as biological replicates.

### HLB Inoculation and Determination of Las Population.

For the HLB infection assay, 2-y-old *CsRD21a*-OE and wild-type control plants were grafted as scions on single branches of individual HLB-symptomatic 8-y-old sweet orange trees in the field at the Science Research Institute of Ganzhou, Jiangxi province, China. The buds of transgenic and control lines were grafted at random positions on the same Las-infected branch. After grafting, sampling was initiated once the transgenic and WT buds had developed to a sufficient size, which generally occurred following a 4-mo growth period. Four months after grafting, leaf midrib samples were collected monthly from both *CsRD21a*-transgenic and WT citrus plants for quantification of Las bacterial populations. In addition, we also grafted HLB-infected citrus buds with similar level of Las populations as scion onto approximately 3-y-old *CsRD21a*-OE and wild-type control plants. Leaf midrib samples were collected monthly from both *CsRD21a*-OE and wild-type citrus plants for quantification of Las bacterial populations. The Las-infected citrus was obtained from an HLB-symptomatic 8-y-old sweet orange tree maintained at the Science Research Institute of Ganzhou, Jiangxi province, China.

DNA was isolated from the leaf midribs monthly after graft inoculation and used to quantify Las populations by Taqman qPCR with primer/probe combination. Las quantification was carried out as follows: DNA was used for qPCR amplification using 16S rRNA primers, the probe HLBp, TaqMan PCR master mix, and SYBR green PCR master mix. The qPCR assays were performed with Light Cycler 480 (Roche) using the SYBR Green PCR Master probe mix (YEASEN) in a 10-*µ*L volume. The data were normalized to the expression of the citrus mitochondrial cytochrome oxidase gene (*COX*). The standard amplification protocol was 95 °C for 10 min followed by 40 cycles at 95 °C for 10 s and 60 °C for 30 s. Las rDNA copy numbers were determined using a standard curve established by Hu et al (72). In brief, DNA concentrations were adjusted to 1 *µ*g/*µ*L using a NanoDrop 1000 spectrophotometer (Thermo Scientific). Serial 10-fold dilutions of Las 16S rDNA were prepared, and these diluted samples were amplified using a TaqMan-based real-time qRT-PCR assay to obtain Ct values. A standard curve was constructed by plotting Ct values on the *x*-axis and log_10_-transformed copy numbers on the y-axis, yielding the regression equation *y* = −0.2672*x* + 10.702 (R^2^ = 0.9992). The primer details can be found in Dataset S3.

### Peptide Synthesis.

The P1-M1-O1 peptide (H-ADPVRRAHHGGRGVVPTIATNRYVPIRHDFNGPYAGLSALYNGSFGEEAHH-OH) was synthesized using the Vapourtec “Peptide Builder” flow peptide synthesizer. 300 mg Wang resin (0.7 mmol/g) was loaded into a reaction vessel and swollen with continuous flow of DMF (5 mL). The peptide column was treated with amino acid (0.3 M, 5 eq.) and oxyma (0.45 M, 7.5 eq.) in N,N-Dimethylformamide (DMF) and 1,3-Diisopropylcarbodiimide (DIC) at 80 °C. After each amino acid coupling the column was washed with DMF and subjected to Fmoc deprotection with 20% piperidine in DMF at 80 °C. Subsequent coupling reactions and deprotections were carried out similarly. Following deprotection of the final amino acid and removal of residual DMF, the peptide was cleaved from the resin, precipitated using Et_2_O, and collected using centrifugation. Peptide purification was achieved using automated flash column chromatography on a Biotage Selekt with a Biotage Sfär C18 D column. Analytical RP-HPLC was performed on an Agilent 1200 using an Agilent eclipse XDB-C18 column. MALDI-MS was performed on a Kratos Analytical Axima-CFR MALDI ToF, using α-cyano-4-hydroxycinnamic acid as the matrix. 97% purity of the P1-M1-O1 peptide was achieved.

### *PR1* Expression Analysis in *N. benthamiana*.

The LasOMP1 protein without its signal peptide was fused to the N-terminal PR1 signal peptide and was coexpressed with *Cs*RD21a or C14 in *N. benthamiana.* GFP and the protease catalytic mutants were used as negative controls. All proteins were expressed using the vector pJK268c in *A. tumefaciens* GV3101 cells, following the same procedure for infiltration in *N. benthamiana* leaves as described above. Alternatively, peptides corresponding to flg22 (Genscript) and LasOMP1 P1-M1-O1 were individually infiltrated in the leaves. RNA was extracted from the agroinfiltrated leaves after 2 d using the RNeasy Plant Mini Kit (Qiagen). Then, 1.0 μg of the extract was digested with 4× gDNA wiper (Vazyme Biotech) to remove gDNA. 5× HiScript II Q RT SuperMix was added to synthesize first-strand complementary DNA (cDNA). The relative expression of *PR1* gene was quantified using RT-qPCR with Taq Pro Universal SYBR qPCR Master Mix (Vazyme) and the CFX Opus 96 Real-Time PCR System (Bio-Rad). RT-qPCR was performed using gene-specific primers (Dataset S3). Equal amounts of cDNA from nine independent biological replicates were analyzed. Three technical replicates were performed for each biological replicate. With the *N. benthamiana β*-Actin gene as the internal reference gene, relative expression levels were calculated using the 2^−ΔΔCT^ method (73).

### AlphaFold Structural Modeling.

All protein structure modeling was performed using AlphaFold3 (74). Predicted models were imaged using ChimeraX (75–77) and added to the “Gram-negative bacteria cell wall” template image sourced from BioRender.com.

## Supplementary Material

Appendix 01 (PDF)

Dataset S01 (XLSX)

Dataset S02 (XLSX)

Dataset S03 (XLSX)

Dataset S04 (XLSX)

## Data Availability

RNA-seq data have been deposited in NCBI Bioproject (BioProject PRJEB82095/) (78).
