# Supplementary material for: A secreted citrus protease cleaves an outer membrane protein of the Huanglongbing pathogen
Source: Proc Natl Acad Sci U S A. 2026 Apr 7;123(15):e2528641123. doi: 10.1073/pnas.2528641123 (PMC13079941; doi:10.1073/pnas.2528641123)
Supplement: Supplementary file 1 — Appendix 01 (PDF) [file pnas.2528641123.sapp.pdf]

Supplementary materials for

A secreted citrus protease cleaves an outer membrane protein of the Huanglongbing pathogen

Alexander J. McClelland[1,#], Bin Hu[2,#], Yuantao Xu[2], Xiaodong Fang[1], Chunxia Wang[3], Benjamin L. Koch[1], Amelia H. Lovelace[1,§], Eva Hawara[4], Yuanchun Wang[3], Zhiqian Pang[3], Agustina De Francesco[4], Suzanne P. van Wier[5], Andrew M. Beekman[5], Amit Levy[3], Nian Wang[3], Renier A. L. van der Hoorn[6], Qiang Xu[2], Wenbo Ma[1,*]

[1] The Sainsbury Laboratory, University of East Anglia, Norwich Research Park, Norwich, NR4 7UH, UK
[2] National Key Laboratory for Germplasm Innovation & Utilization of Horticultural Crops, Huazhong Agricultural University, Wuhan, 430070, China
[3] Citrus Research and Education Center, Department of Plant Pathology, Microbiology and Cell Science, University of Florida/IFAS, Lake Alfred, Florida, 33850, USA
[4] Department of Microbiology and Plant Pathology, University of California Riverside, Riverside, CA 92521, USA
[5] School of Chemistry, Pharmacy and Pharmacology, University of East Anglia, Norwich, NR4 7TJ, United Kingdom
[6] Plant Chemetics Laboratory, Department of Biology, University of Oxford, South Parks Road, Oxford, OX1 3RB, UK

[#] Equal contribution: Alexander J. McClelland and Bin Hu

* Corresponding author: Wenbo Ma: The Sainsbury Laboratory, University of East Anglia, Norwich Research Park, Norwich, NR4 7UH, UK

[§] Current address: Sustainable Perennial Crops Laboratory, Agricultural Research Service, United States Department of Agriculture, Beltsville, MD, 20705, USA

Email: wenbo.ma@tsl.ac.uk

This file includes:

Figures S1 to S10

Legends for Dataset S1 to S4

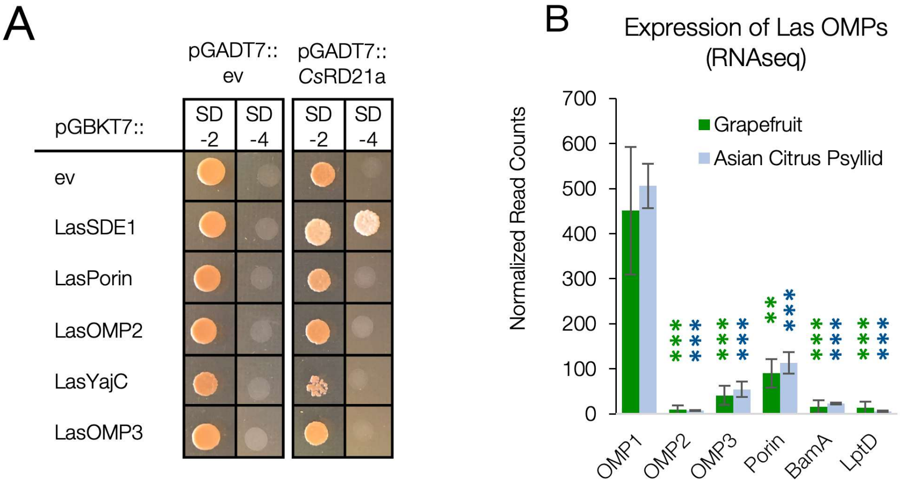

Figure S1. *Cs*RD21a does not interact with other Las OMPs, which exhibit lower expression than LasOMP1.

(**A**) Yeast-two-hybrid assay examining interaction of *Cs*RD21a (prey) with Las OMPs (bait) with LasSDE1 as the positive control and empty vector (ev) as the negative control. LasYajC, an inner membrane protein, was also examined. SD-2 medium lacks leucine and tryptophan and was used to select the co-transformed colonies. One colony with OD_{600} = 1.0 was plated for each co-transformation in yeast on both SD-2 and SD-4, which lacks leucine, tryptophan, adenine, and histidine. All proteins were expressed without their signal peptides. (**B**) Expression of Las OMPs in infected grapefruit midribs (n=6) and the Asian citrus psyllid (n=4) represented by normalized read counts using publicly available data (32). Error bars indicate standard deviation. Asterisks (*) represent degrees of significance as determined by two-tailed T-test between each gene and LasOMP1, where ** signifies $p < 0.01$ and *** signifies $p < 0.001$. Green and blue asterisks represent significance for the grapefruit and Asian citrus psyllid samples, respectively.

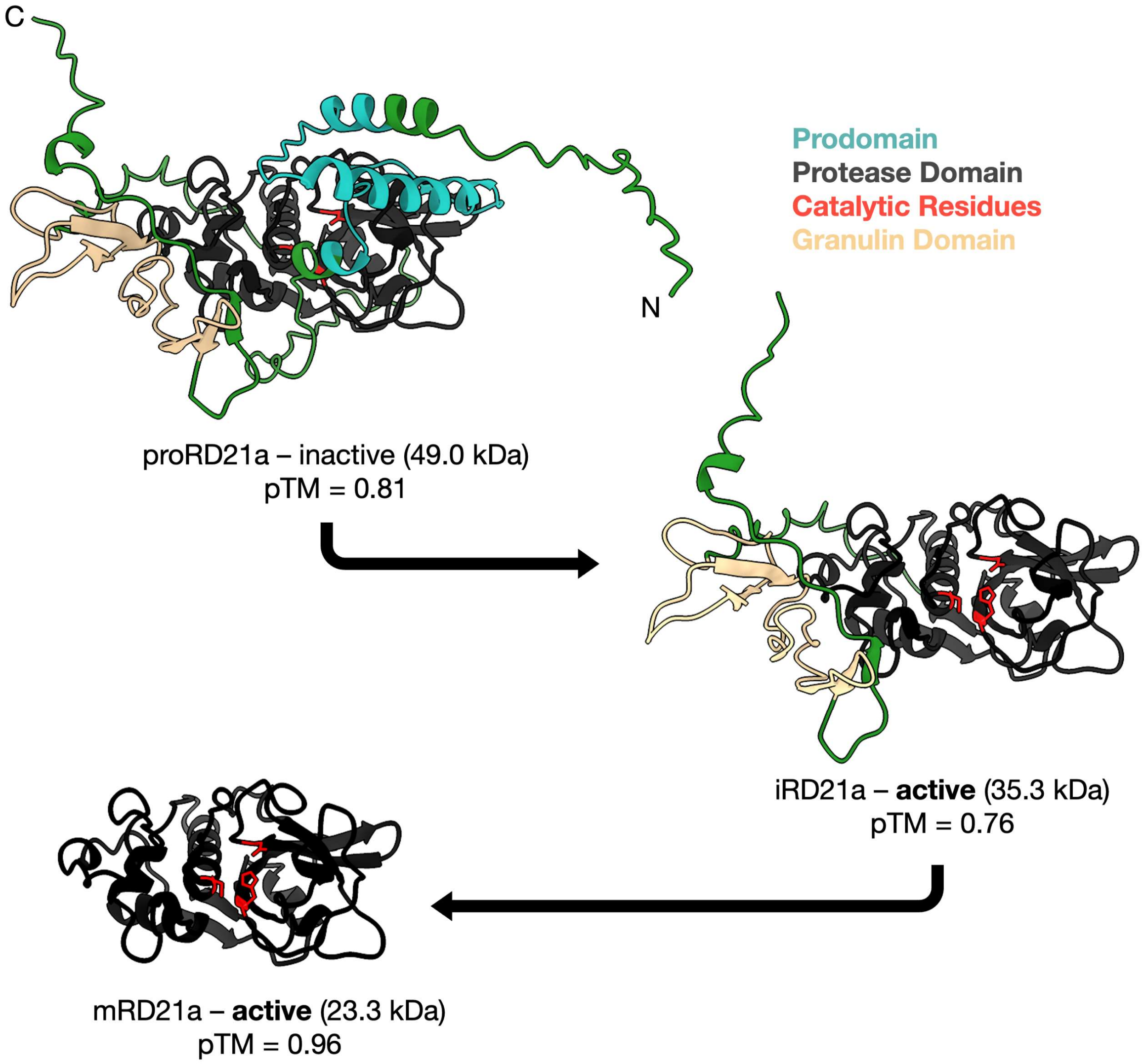

Figure S2. Processing of *Cs*RD21a in planta.

*Cs*RD21a *s*tructures were predicted with AlphaFold3 and visualized using ChimeraX. RD21a proteins have an N-terminal signal peptide (excluded from this model), an autoinhibitory prodomain (blue), a catalytic protease domain (black), three catalytic residues (red), and a C-terminal granulin domain (tan). proRD21a (shown at the top) is generated when the N-terminal signal peptide has been removed, but the enzyme is inactive due to the presence of the autoinhibitory prodomain. iRD21a is an active intermediate in which the prodomain has been removed, exposing the catalytic residues. mRD21a is a further processed, active enzyme that has had its granulin domain removed. The protein molecular weights (in kDa) and AlphaFold3 confidence scores (in pTM) are shown. N- and C-termini are labeled on proRD21a.

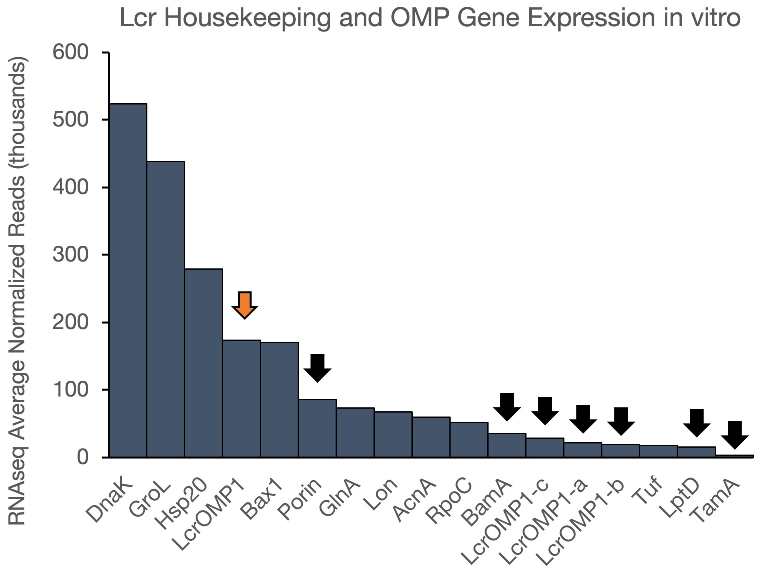

Figure S3. LcrOMP1 is highly expressed.

Expression levels, represented by average normalized reads (two replicates), of genes encoding OMPs (marked with black arrows, LcrOMP1 with an orange arrow) in Lcr grown in artificial medium as determined by RNA-seq. Some of the most highly expressed housekeeping genes are used as a comparison. All OMPs, except for TamA, are in the top 17% of highest expressed genes in the dataset (Table S2).

A

	Amino acid % similarity to LasOMP1
LasOMP1	100.00
LafOMP1	49.51
LsoOMP1	45.32
LamOMP1	41.33
LcrOMP1	35.98
LcrOMP1-a	28.95
LcrOMP1-b	27.75
LcrOMP1-c	29.63

B

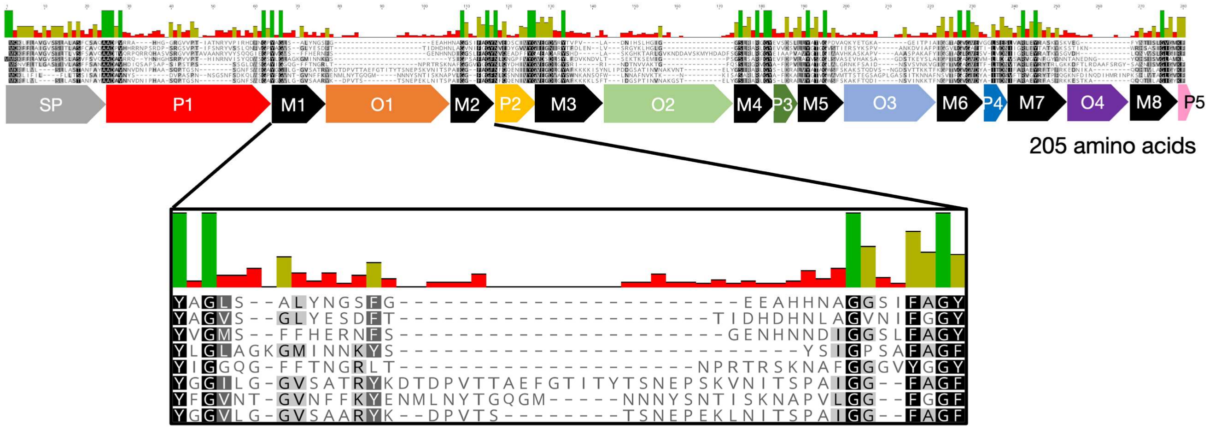

Figure S4. Sequence comparison of OMP1 homologs in *Liberibacter* spp.

(**A**) Amino acid sequence similarity as determined by MUSCLE alignment (56) of OMP1 homologs. (**B**) MUSCLE alignment of OMP1 homologs mapped to the domain structure of LasOMP1. Conservation levels are displayed for each amino acid (green = 100% conserved, green-brown = 30-99% conserved, red = 0-29% conserved). The inset zooms in the M1-O1-M2 region. Sequences in the alignment are presented in the same order as panel A.

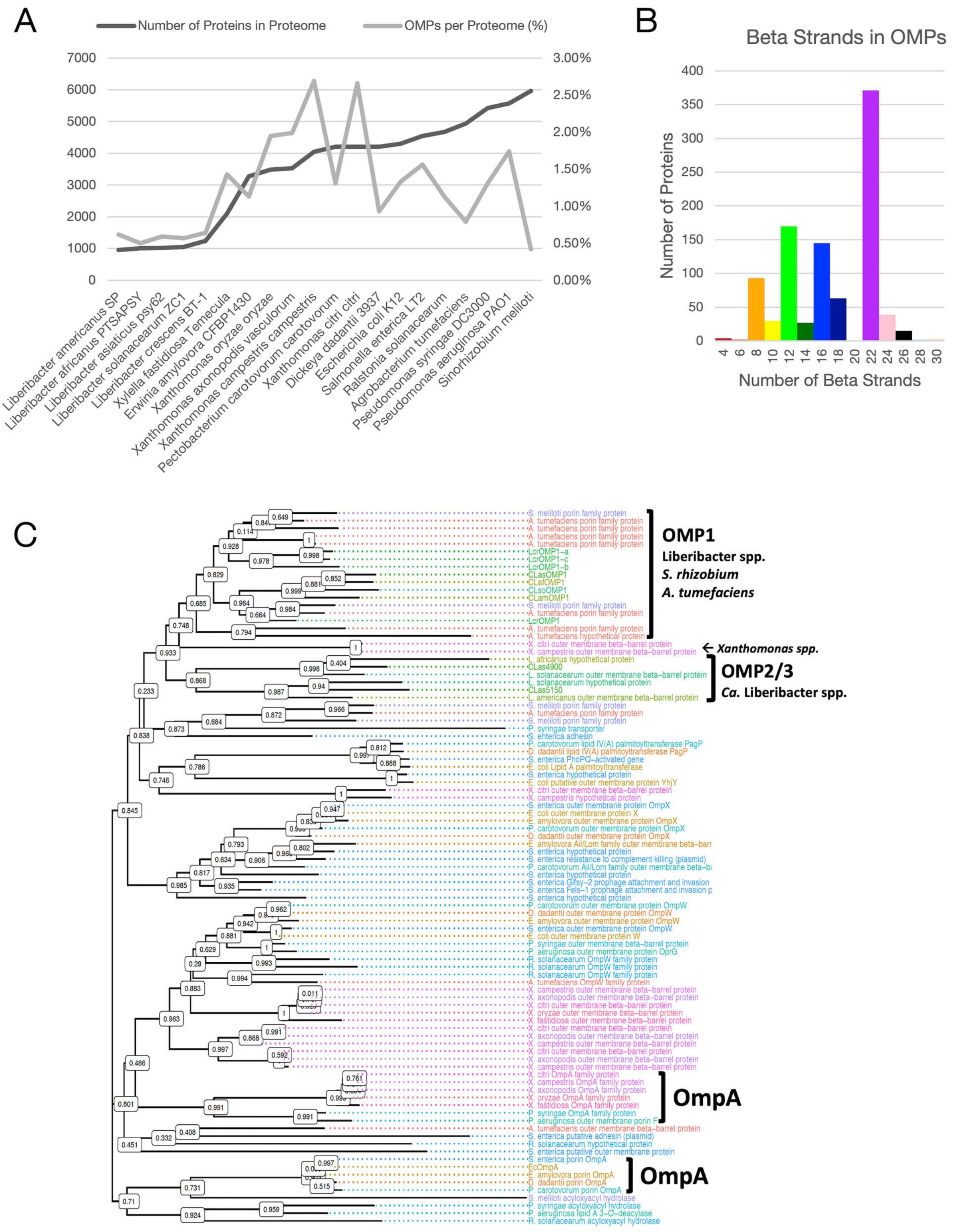
A
Number of Proteins in Proteome
OMPs per Proteome (%)
7000
6000
5000
4000
3000
2000
1000
0
3.00%
2.50%
2.00%
1.50%
1.00%
0.50%
0.00%
Liberibacter americanus SP
Liberibacter africanus PTSAPSY
Liberibacter asiaticus psy62
Liberibacter solanacearum ZC1
Liberibacter crescens BT-1
Xylella fastidiosa Temecula
Erwinia amylovora CFBP1430
Xanthomonas oryzae oryzae
Xanthomonas axonopodis vasculorum
Xanthomonas campestris campestris
Pectobacterium carotovorum carotovorum
Xanthomonas citri citri
Dickeya dadantii 3937
Escherichia coli K12
Salmonella enterica LT2
Ralstonia solanacearum
Agrobacterium tumefaciens
Pseudomonas syringae DC3000
Pseudomonas aeruginosa PAO1
Sinorhizobium meliloti
B
Beta Strands in OMPs
Number of Proteins
400
350
300
250
200
150
100
50
0
4
6
8
10
12
14
16
18
20
22
24
26
28
30
Number of Beta Strands
C
OMP1
Liberibacter spp.
S. rhizobium
A. tumefaciens
Xanthomonas spp.
OMP2/3
Ca. Liberibacter spp.
OmpA
OmpA

Figure S5. OMP analyses in 20 representative Gram-negative bacteria.

(**A**) Number of proteins as well as the percentage of predicted OMPs in each bacterium. (**B**) Number of OMPs in each category based on their number of transmembrane beta sheets as determined by the DeepTMHMM output (31). (**C**) Phylogenetic tree of the 8-stranded beta barrel OMPs predicted from the 20 bacteria. A complete list of the predicted OMPs is in Dataset S2. This tree was generated from a MUSCLE alignment (56) and visualized using ggtree (60). Clades that have OMPs identified from Liberibacter, as well as the well-studied OmpA, are highlighted.

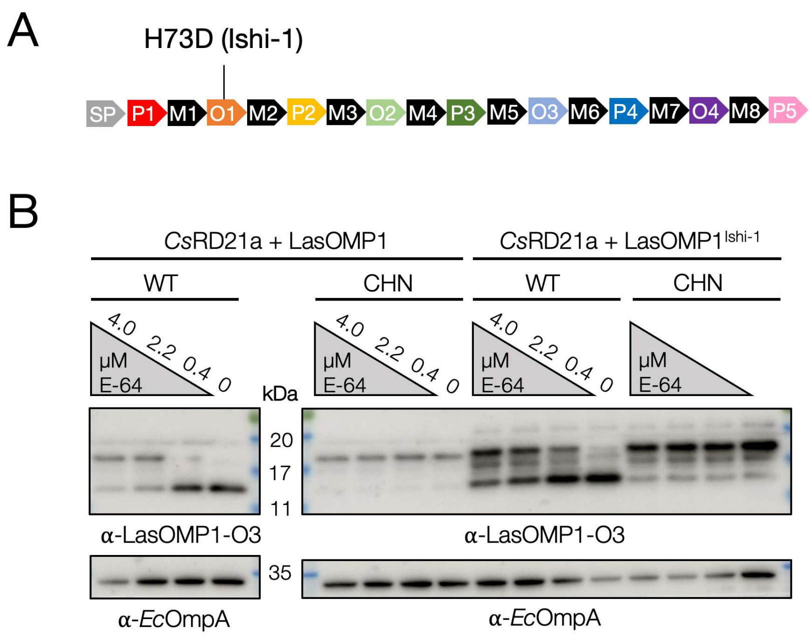

Figure S6. *Cs*RD21a cleaves LasOMP1$^{Ishi\text{-}1}$.

(**A**) Domain structure of LasOMP1 (strain Ishi-1) depicting a non-synonymous mutation found in the O1 domain. (**B**) Semi-in vitro protease cleavage assay of *Cs*RD21a with LasOMP1 or the LasOMP1$^{Ishi\text{-}1}$ variant. E-64 or DMSO-treated apoplastic fluid containing *Cs*RD21a wild-type (WT) or CHN mutant (CHN) was incubated with LasOMP1 or the LasOMP1$^{Ishi\text{-}1}$ variant from *E. coli* membrane fractions, and *Ec*OmpA was used as a loading control. Both OMPs were detected via Western blot using protein-specific antibodies.

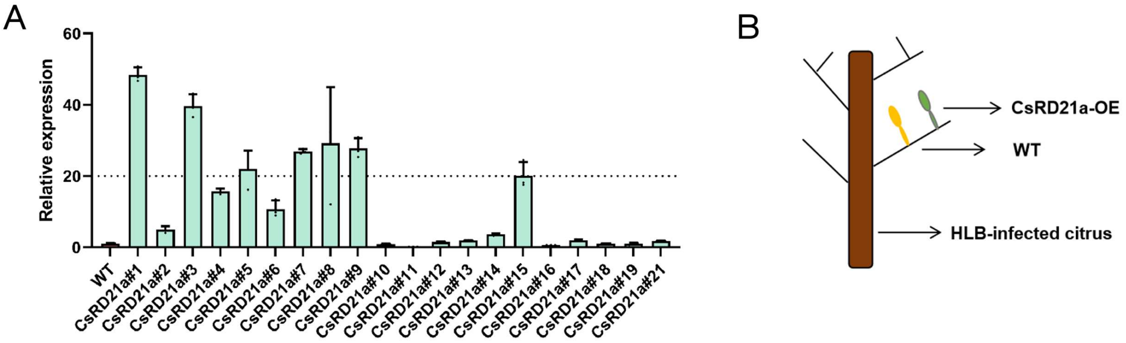

Figure S7. Confirmation of *CsRD21a* overexpression in transgenic sweet orange plants.

(**A**) Relative expression of *CsRD21a* in 20 independent *CsRD21a*-overexpressing (OE) lines compared to wild-type (WT) plants. Expression was determined by RT-qPCR and normalized to the citrus *β-Actin* gene. Data represent means ± standard error (SE) from three biological replicates. Lines 1, 3, 5, 7, 8, and 15 were used for subsequent Las infection assays. (**B**) Schematic diagram showing the grafting of *Cs*RD21a-OE or WT scions on the same branch of HLB-infected citrus.

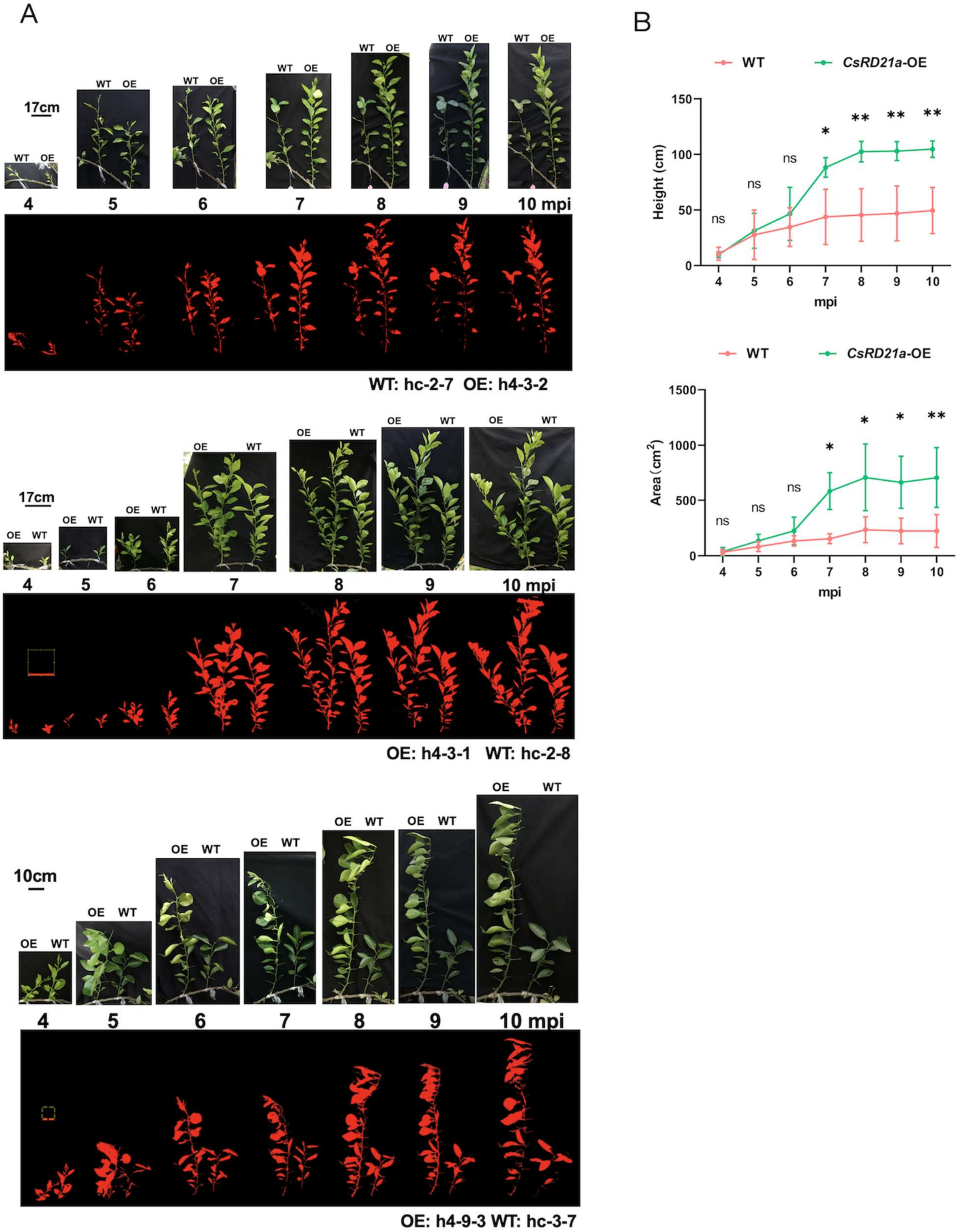
A
17cm
WT OE
4
5
6
7
8
9
10 mpi
WT: hc-2-7 OE: h4-3-2
17cm
OE WT
OE: h4-3-1 WT: hc-2-8
10cm
OE: h4-9-3 WT: hc-3-7
B
WT
CsRD21a-OE
Height (cm)
mpi
ns
*
**
Area (cm2)

Figure S8. The growth of scions that overexpress *CsRD21a* is less restricted by Las infection.

(**A**) Images showing wildtype (WT) and *CsRD21a*-OE scions grafted on HLB-diseased rootstock during six months from 4-10 months post inoculation (mpi). Three independent transgenic lines were analyzed. The images were converted to 8-bit grayscale images then binary images (in red). (**B**) Heights (top panel) and leaf areas (lower panel) of each scion were measured over the monitoring period. Asterisks indicate significant differences based on Student's t-test: ns = no significance, * = $p < 0.05$, and ** = $p < 0.01$.

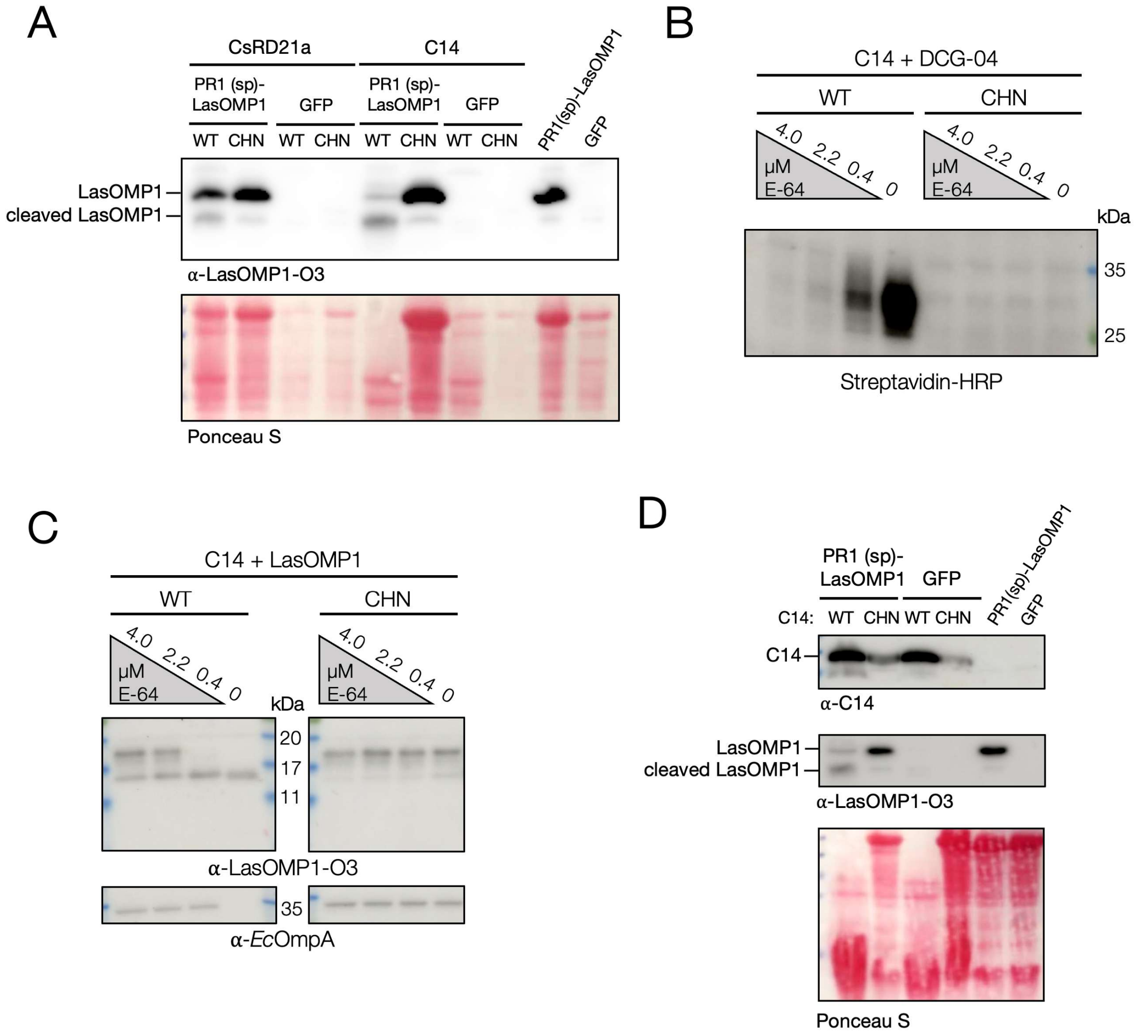

Figure S9. Tomato RD21a (C14) cleaves LasOMP1.

(**A**) Western blot depicting LasOMP1 cleavage by *Cs*RD21a or C14. LasOMP1 fused to the signal peptide (SP) from PR1, instead of its native SP, was co-expressed with wild-type (WT) or catalytic mutant (CHN) *Cs*RD21a or C14 in *N. benthamiana*. Green fluorescent protein (GFP) was used as a negative control. (**B**) Western blot detection of wild-type (WT) or catalytically dead (CHN) C14 in apoplastic fluid (AF) using activity-based protein profiling (ABPP), where a concentration gradient of the cysteine protease inhibitor E-64 was incubated with the AF prior to DCG-04 labeling. Western blot detection of DCG-04-bound proteases was performed using Streptavidin-HRP. (**C**) LasOMP1 is cleaved by C14 in the semi-in vitro assay. LasOMP1 was expressed in *E. coli* and the resulting membrane fractions were incubated with E-64- or DMSO-treated, C14-containing apoplastic fluid. *Ec*OmpA could also be cleaved by C14, but only in the absence of E-64. (**D**) Confirmation of C14 expression when the WT protein or CHN mutant is co-expressed with LasOMP1 or GFP in *N. benthamiana*. These samples were included in the RT-qPCR analysis in Fig. 5E.

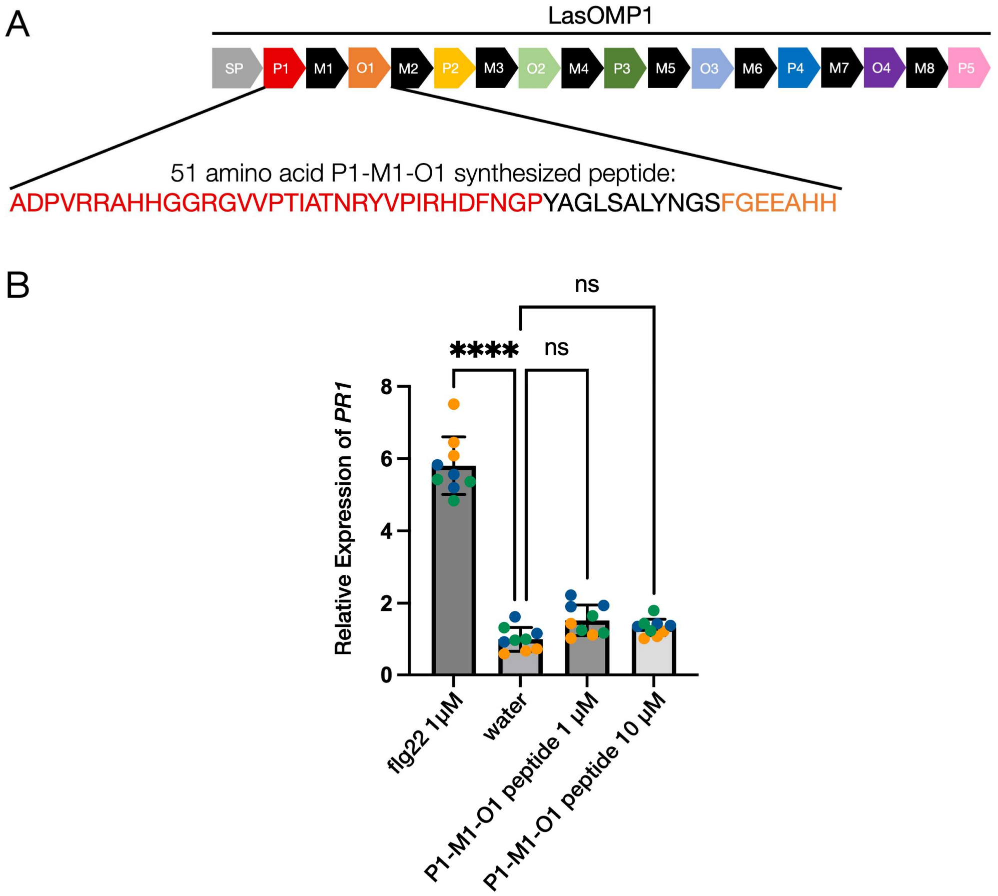

Figure S10. A putative RD21a-produced N-terminal peptide of LasOMP1 does not activate *PR1* expression.

(**A**) A schematic showing the 51-amino acid peptide corresponding to the P1-M1-O1 region of LasOMP1 that was synthesized. (**B**) The P1-M1-O1 peptide (1 μM and 10 μM) was infiltrated into 4-week-old *N. benthamiana* plants, and *PR1* expression was examined at 6 hours post-infiltration by RT-qPCR. The flg22 peptide (1 μM) and water were also infiltrated as positive and negative controls, respectively. Data include three *N. benthamiana* leaves from three biological replicates (as shown by green, blue, and yellow dots). One-way ANOVA was performed to determine statistical significance, where **** signifies $p < 0.0001$ and ns = not significant.

Supplementary Datasets

Dataset S1. HMM-predicted OMPs in Liberibacter.

Beta barrel OMPs predicted by both hmmsearch and DeepTMHMM in representative Liberibacter strains: Las (strain psy62), Laf (strain PTSAPSY), Lso (strain ISR100), Lam (strain PW_SP), and Lcr (strain BT-1). Annotations are derived from NCBI reference genomes for each strain, and clusters are colored based on Figure 3A.

Dataset S2. Beta barrel OMPs predicted by both DeepTMHMM and hmmsearch in Gram-negative bacteria.

Dataset S3. Primers used in this study.

Dataset S4. Normalized counts of 1,388 genes in *Liberibacter crescens* BT-1 transcriptome for duplicate bacterial samples collected from liquid culture.

Genes are ordered based on average normalized counts in descending order. The normalized counts were calculated using counts function in DESeq2 v.1.30.1 R package. Gene, gene names, old locus names, and description were taken from NCBI.
